# Trichlorobenzene (all isomers): MAK Value Documentation, addendum – Translation of the German version from 2022

**DOI:** 10.34865/mb1200248isme10_2ad

**Published:** 2025-06-30

**Authors:** Andrea Hartwig, MAK Commission

**Affiliations:** 1 Institute of Applied Biosciences. Department of Food Chemistry and Toxicology. Karlsruhe Institute of Technology (KIT) Adenauerring 20a, Building 50.41 76131 Karlsruhe Germany; 2 Permanent Senate Commission for the Investigation of Health Hazards of Chemical Compounds in the Work Area. Deutsche Forschungsgemeinschaft, Kennedyallee 40, 53175 Bonn, Germany. Further information: Permanent Senate Commission for the Investigation of Health Hazards of Chemical Compounds in the Work Area | DFG

**Keywords:** 1,2,3-Trichlorbenzol, 1,2,4-Trichlorbenzol, 1,3,5-Trichlorbenzol, Porphyrinurie, maximale Arbeitsplatzkonzentration, MAK-Wert, Kanzerogenität, Mutagenität, Toxizität, Metabolismus, 1,2,3-trichlorobenzene, 1,2,4-trichlorobenzene, 1,3,5-trichlorobenzene, porphyrinuria, maximum workplace concentration, MAK value, carcinogenicity, mutagenicity, toxicity, metabolism

## Abstract

The German Senate Commission for the Investigation of Health Hazards of Chemical Compounds in the Work Area (MAK Commission) has re-evaluated the occupational exposure limit value (maximum concentration at the workplace, MAK value) of trichlorobenzene isomers (1,2,3-trichlorobenzene [87-61-6], 1,2,4-trichlorobenzene [120-82-1] and 1,3,5-trichlorobenzene [108-70-3]) considering all toxicological end points. Relevant studies were identified from a literature search and also unpublished study reports were used. The critical effects of the trichlorobenzenes are adverse effects on the livers of mice and rats and on the kidneys of rats. These effects were more pronounced after exposure to 1,2,4-trichlorobenzene with the rat being the most sensitive species. However, as the isomers all have a similar primary toxicological mode of action and follow similar metabolic pathways, they have been evaluated together. After exposure to 1,2,4-trichlorobenzene, liver toxicity was manifest in rats and mice as increased liver weights and histological changes in the liver were preceded by an induction of metabolic enzymes. The most sensitive end point, a disruption of porphyrin biosynthesis, was observed in a 90-day inhalation study in rats with a NOAEC of 3 ml/m^3^. On this basis, the maximum concentration at the workplace (MAK value) has been set at 0.5 ml/m^3^. As the critical effect of the trichlorobenzenes is systemic, Peak Limitation Category II has been assigned with an excursion factor of 2. The trichlorobenzenes are not genotoxic. The neoplasms detected in rats and mice in carcinogenicity studies with 1,2,4-trichlorobenzene are considered to be of no human relevance. In rats, the NOAEC for developmental toxicity was 840 mg/m^3^ for 1,2,3-trichlorobenzene and 1,3,5-trichlorobenzene and 420 mg/m^3^ for 1,2,4-trichlorobenzene. The NOAEC for perinatal toxicity was 54 mg/m^3^ for 1,2,4-trichlorobenzene. As no teratogenicity was observed and the margins between the NOAECs and the MAK value are sufficiently large, the trichlorobenzenes have been assigned to Pregnancy Risk Group C. Data for skin sensitizing effects are limited to experiments performed with 1,2,3-trichlorobenzene that confirmed the induction of sensitizing effects. Therefore, only 1,2,3-trichlorobenzene has been designated with “Sh”. There are no data for sensitization of the respiratory tract. According to skin absorption models, 1,2,4-trichlorobenzene is expected to be taken up via the skin in toxicologically relevant amounts. Therefore, all trichlorobenzenes remain designated with “H”.

**Table:** 

**MAK value (2021)**	**0.5 ml/m^3^ (ppm) ≙ 3.8 mg/m^3^**
**Peak limitation (2021)**	**Category II, excursion factor 2**
	
**Absorption through the skin (1996)**	**H**
**Sensitization (2021)**	**1,2,3-trichlorobenzene: Sh** **1,2,4-trichlorobenzene and 1,3,5-trichlorobenzene: –**
**Carcinogenicity**	**–**
**Prenatal toxicity (2021)**	**Pregnancy Risk Group C**
**Germ cell mutagenicity**	**–**
	
**BAT value**	**–**
	
Synonyms	1,2,3-trichlorobenzene: *vic*-trichlorobenzene 1,2,4-trichlorobenzene: *unsym*-trichlorobenzene 1,3,5-trichlorobenzene: *sym*-trichlorobenzene
Chemical name (IUPAC)	1,2,3-trichlorobenzene 1,2,4-trichlorobenzene 1,3,5-trichlorobenzene
CAS number	1,2,3-trichlorobenzene: 87-61-6 1,2,4-trichlorobenzene: 120-82-1 1,3,5-trichlorobenzene: 108-70-3
Structural formula	1,2,3-trichlorobenzene: 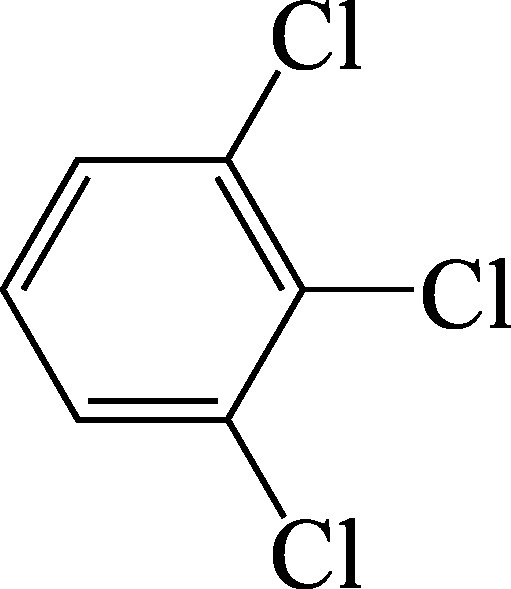
	1,2,4-trichlorobenzene: Structural formula of 1,2,4-trichlorobenzene. 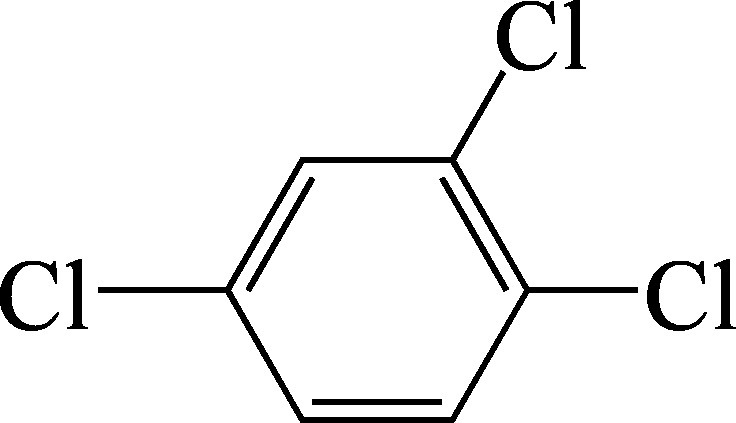
	1,3,5-trichlorobenzene:Structural formula of 1,3,5-trichlorobenzene. 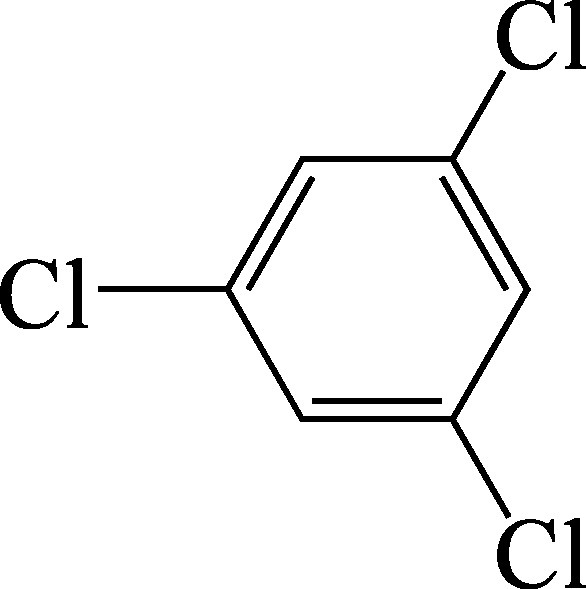
Molecular formula	>C_6_H_3_Cl_3_
Molar mass	181.45 g/mol
Melting point	1,2,3-trichlorobenzene: 53 °C (IFA 2020 a)1,2,4-trichlorobenzene: 17 °C (IFA 2020 b)1,3,5-trichlorobenzene: 63 °C (IFA 2020 c)
Boiling point at 1013 hPa	1,2,3-trichlorobenzene: 221 °C (IFA 2020 a)1,2,4-trichlorobenzene: 213 °C (IFA 2020 b)1,3,5-trichlorobenzene: 209 °C (IFA 2020 c)
Density at 20 °C	1,2,3-trichlorobenzene: 1.69 g/cm^3^ (IFA 2020 a)1,2,4-trichlorobenzene: 1.46 g/cm^3^ (IFA 2020 b)1,3,5-trichlorobenzene: 1.46 g/cm^3^ (NCBI 2020 c)
Vapour pressure at 25 °C	1,2,3-trichlorobenzene: 0.28 hPa, determined experimentally (NCBI 2020 a)1,2,4-trichlorobenzene: 0.61 hPa, determined experimentally (NCBI 2020 b)1,3,5-trichlorobenzene: 0.32 hPa, determined experimentally (NCBI 2020 c)
log K_OW_	1,2,3-trichlorobenzene: 4.05 (NCBI 2020 a)1,2,4-trichlorobenzene: 4.02 (NCBI 2020 b)1,3,5-trichlorobenzene: 4.19 (NCBI 2020 c)
Solubility in water at 25 °C	1,2,3-trichlorobenzene: 18 mg/l (NCBI 2020 a)1,2,4-trichlorobenzene: 49 mg/l (NCBI 2020 b)1,3,5-trichlorobenzene: 6.01 mg/l (NCBI 2020 c)
**1 ml/m^3^ (ppm) ≙ 7.529 mg/m^3^**	**1 mg/m^3^ ≙ 0.133 ml/m^3^ (ppm)**
	
Hydrolytic stability	no data
Production	by reaction of liquid benzene with chlorine gas in the presence of a catalyst (Lewis acid, for example iron chloride) ATSDR 2014)
Uses	1,2,4-trichlorobenzene as a solvent; 1,2,3-trichlorobenzene and 1,3,5-trichlorobenzene as intermediates; outside of the European Union, 1,2,4-trichlorobenzene is produced in larger quantities than the other 2 isomers ATSDR 2014)

This addendum is based on reviews of toxicological data (ATSDR 2014; EC 2003; US EPA 2009 a, b) and other sources. There are also publicly available REACH registration data (ECHA 2014, 2018 a).

In the European Union, the use of 1,2,4-trichlorobenzene as a substance or in mixtures in concentrations greater than 0.1% is prohibited except if used as an intermediate or in closed systems or for the production of 1,3,5-tri­amino-2,4,6-trinitrobenzene (ECHA n.d.). 1,3,5-Trichlorobenzene has not been registered for any use in Europe (ECHA 2018 b).

Documentation for all 3 trichlorobenzenes was published in 1990 (Henschler 1992), followed by an addendum in 1996 that evaluated the carcinogenicity of 1,2,4-trichlorobenzene (Greim 2000). Further addenda reviewed the data for 1,2,3-trichlorobenzene and 1,3,5-trichlorobenzene with respect to absorption through the skin (Greim 1998, available in German only), peak limitation (Greim 2002 b, available in German only) and developmental toxicity (Greim 2007, available in German only). Cited unpublished toxicological studies from companies have been made available to the Commission.

In 2016, the Commission began using a revised approach for assessing substances with a MAK value based on systemic effects that was derived from findings from inhalation studies in animals or studies with volunteers at rest; this approach takes into account that the respiratory volume at the workplace is higher than under experimental conditions. However, the approach cannot be used for gases or vapours with a blood:air partition coefficient < 5 (see List of MAK and BAT Values, Section I b and I c; DFG 2020). By applying the formula of Buist et al. (2012), the blood:air partition coefficients for 1,2,3-trichlorobenzene, 1,2,4-trichlorobenzene and 1,3,5-trichlorobenzene were calculated to be 224, 270 and 247, respectively. This addendum evaluates whether the MAK values for 1,2,3-trichlorobenzene and 1,3,5-trichlorobenzene need to be amended as a result of the higher respiratory volume at the workplace and whether it is possible to derive a MAK value for 1,2,4-trichlorobenzene.

## 1 Toxic Effects and Mode of Action

The critical effects are the adverse effects on the liver and kidneys caused by subchronic or chronic inhalation exposure and oral exposure. In addition, effects on the adrenal glands were observed that led to subsequent effects on the blood and thyroid glands. 1,2,4-Trichlorobenzene induces stronger effects than the other 2 isomers. After rats and mice were exposed to 1,2,4-trichlorobenzene, reduced body weights and increased organ weights in addition to histological changes in the liver were observed. Male rats had increased kidney weights. Oxidative, reductive and conjugating enzymes were induced at an early stage of the adverse reaction to the trichlorobenzenes. In rats, the species which is especially sensitive, 1,2,4-trichlorobenzene affects, among others, enzymes of haem biosynthesis in the liver, particularly δ-aminolaevulinate synthetase and haem oxygenase, leading to a disruption of porphyrin metabolism. This was detected by an increased excretion of uroporphyrin and coproporphyrin in rats exposed for 90 days to 1,2,4-trichlorobenzene concentrations of 10 ml/m^3^ and above. This effect is the most sensitive end point after inhalation exposure. Other adaptive and adverse effects such as cell hypertrophy and increased cell proliferation, increased organ weights and necrosis in the liver and kidneys as well as reduced body weights were found only at higher concentrations of 25 ml/m^3^ and above.

In valid studies, no evidence of a mutagenic or clastogenic potential was found for the 3 trichlorobenzenes.

Liver adenomas and liver carcinomas were observed in female and male B6C3F1 mice, a strain that is highly susceptible for these effects, at 1,2,4-trichlorobenzene doses of 127 and 100 mg/kg body weight and day, respectively, and above. Their induction was attributed to the tumour-promoting effects of the substance and to the toxic effects on the liver that preceded their development. The tumour incidences in rats were not increased with statistical significance.

The trichlorobenzenes caused slight to moderate reversible irritation of the skin and eyes of rabbits and guinea pigs. Only at higher concentrations or after longer or repeated exposure, damage became manifest.

No teratogenicity or toxic effects on development were observed.

A valid local lymph node assay (LLNA test) demonstrated that 1,2,3-trichlorobenzene has skin sensitizing potential. There are still no findings for skin sensitizing effects of 1,2,4-trichlorobenzene and 1,3,5-trichlorobenzene in humans or animals or from in vitro studies. There are no data available for sensitizing effects of the 3 isomers on the respiratory tract.

## 2 Mechanism of Action

Animal studies reported that exposure to the trichlorobenzenes caused adverse effects in the liver and kidneys (see Section 5). Rats are the species most sensitive to liver toxicity; males are more susceptible than females. The adverse effects are attributed to the induction of enzymes and the formation of reactive intermediates; in rats, the first noticeable sign of these effects is the disruption of porphyrin metabolism (Dow Chemical Company 1977). Overall, the trichlorobenzenes have a complex mechanism of action that is not yet fully understood. The processes and causative factors that give rise to these effects are described below.

### Enzyme activities

In the liver, the trichlorobenzenes induce cytochrome P450 (CYP) enzymes, cytochrome-*c*-reductase, acetanilide hydrolase and acetanilide esterase, procaine esterase and aryl esterase, 4-dimethylaminoantipyrine-*N*-demethylase and aldrine epoxidase. Of the 3 isomers, 1,2,4-trichlorobenzene is the strongest inducer (Henschler 1992). Recent in vitro studies using HepaRG liver cells likewise found evidence of the modulation of CYP activities: exposure to 1,2,4-trichlorobenzene modulated the expression of the genes *CYP1A1*, *CYP2E1*, *CYP3A5* and *CYP7A1* whereas exposure to 1,2,3-trichlorobenzene induced *CYP1A1* and *CYP1A2* (US EPA 2020 a, b). The trichlorobenzenes, and particularly 1,2,4-trichlorobenzene, induced oxidative, reductive and conjugative enzymes mainly in the liver. This is considered one cause of the liver toxicity of the substances (ATSDR 2014; US EPA 2009 b).

### Porphyrin metabolism

A disturbance in porphyrin metabolism can be determined via the increased excretion of the intermediate products of haem biosynthesis such as uroporphyrin and coproporphyrin with the urine. This is caused by the disruption of haem biosynthesis in the liver and bone marrow. The enzymes that are responsible for controlling the rate at which haem is metabolised are δ-aminolaevulinate synthetase and haem oxygenase. The production of haem increases with the activation of the first enzyme, leading to a disruption in metabolism and the increased excretion of intermediates. In vivo studies found that 1,2,4-trichlorobenzene induces δ-aminolaevulinate synthetase in Wistar rats (Ariyoshi et al. 1975 a, b; Kato et al. 1988, 1993; Kato and Kimura 2002). A metabolite of 1,2,4-trichlorobenzene, 2,3,5-trichlorophenyl methyl sulfone, plays a major role in this, while the parent substance itself induces haem oxygenase, which likewise leads to the disruption of haem metabolism (Kato et al. 1988, 1993; Kato and Kimura 2002). δ-Aminolaevulinate synthetase is regulated in the liver by feedback inhibition via haem (Besur et al. 2014). If haem is metabolized more rapidly due to the induction of haem oxygenase, this may further promote an increase in δ-aminolaevulinate synthetase activity and interference in porphyrin metabolism. In rats, porphyrin metabolism was already disrupted after inhalation exposure for 3 months to a 1,2,4-trichlorobenzene concentration of 10 ml/m^3^; there was no evidence of other effects on the organism. Therefore, this disruption is the most sensitive end point of exposure to 1,2,4-trichlorobenzene. There is no evidence that 2,3,5-trichlorophenyl methyl sulfone, which is assumed to be the critical metabolite, forms from 1,2,3-trichloro­benzene and 1,3,5-trichlorobenzene. It was found that exposure to 1,2,3-trichlorobenzene and 1,3,5-trichlorobenzene leads to the increased induction of δ-aminolaevulinate synthetase activity in rats; however, the increase was not statistically significant (Ariyoshi et al. 1975 a).

### Organ toxicity

In addition to the disruption of porphyrin metabolism, effects on other enzymes lead to cellular changes and cellular damage in the liver as well as inflammatory effects in this organ. This gives rise to secondary toxic effects such as anaemia and effects on the thyroid gland caused by alterations in hepatic thyroxine metabolism (US EPA 2009 b). Furthermore, in vitro studies demonstrated that 1,2,4-trichlorobenzene is able to compete with thyroxine at the binding site of TTR (transthyretin). In comparison with other investigated chlorinated hydrocarbons such as pentachlorophenol, this ability to compete increases with the extent of chlorine substitution (den Besten et al. 1991 b). Adverse effects on the kidneys are caused by cytotoxicity, probably α2u-globulin nephropathy; however, this mechanism is not relevant for humans (see Section “Cytotoxicity and tumour promotion”).

### Arene oxides and quinones

Arene oxides may form during the metabolism of all 3 trichlorobenzene isomers. They are strong electrophiles and thus highly reactive. Arene oxides then form quinones and hydroquinones that interact further with proteins and other cell components, possibly impairing their functionality. Therefore, these intermediates may be another possible cause of the adverse effects on the liver and at other target sites (ATSDR 2014; US EPA 2009 b). In vitro studies found that 1,2,4-trichlorobenzene forms quinones and that these bind to microsomal proteins (den Besten et al. 1991 a; see Section 5.6). Furthermore, in vivo studies carried out with male ddY mice that were given a single 1,2,4-trichlorobenzene dose of 1.5 mmol/kg body weight by intraperitoneal injection (equivalent to about 270 mg/kg body weight) at the age of 6 weeks found evidence of the formation of quinones. Liver toxicity (alanine aminotransferase activity in serum) was significantly reduced by pre-treating the animals with 0.5% BHA (butylated hydroxyanisole) administered with the feed. As the toxic effects of chloroform on the liver were not reduced by treatment with BHA, the authors concluded that, unlike the damage induced by chloroform, quinones play a part in the mechanism of action of 1,2,4-trichlorobenzene. This assumption is supported mainly by the fact that BHA activates glutathione-*S*-transferases, epoxide hydrolases and glucuronosyl transferases in the liver and is actively involved in the detoxification of quinones as an antioxidant (ATSDR 2014; Mizutani and Miyamoto 1999).

### Cytotoxicity and tumour promotion

In a 104-week feeding study, adenomas and carcinomas developed only in the liver of B6C3F1 mice, a mouse strain that is susceptible to this effect, at 1,2,4-trichlorobenzene doses of 100 and 127 mg/kg body weight and day, respectively, and above. The animals in these dose groups already exhibited marked adverse, substance-induced effects on the liver such as centrilobular hepatocytomegaly and increased relative and absolute liver weights or cell changes during the study period. Carcinogenicity studies that investigated 1,4-dichlorobenzene likewise found hepatocellular carcinomas only in B6C3F1 mice at hepatotoxic doses. It has been suggested that chlorohydroquinones and their glutathione conjugates are involved in the carcinogenicity of 1,4-dichlorobenzene via the formation of reactive oxygen species (ROS) (Hartwig and MAK Commission 2019). The chlorinated biphenyls are another group of substances that use this process; they induce much stronger adverse effects than the trichlorobenzenes. Studies carried out in rats with these substances demonstrated that the formation of quinones leads to the development of oxygen radicals. These, in turn, form hydrogen peroxide via a process catalysed by superoxide dismutase. Hydrogen peroxide influences the mitotic signal chain in liver cells, resulting in the proliferation of previously initiated tumour cells (Brown et al. 2007). In theory, the quinones of the trichlorobenzenes are likewise able to form ROS. However, according to den Besten et al. (1991 a, b), redox cycling is not a mechanism that occurs extensively with chlorinated biphenyls because increased lipid peroxidation as a possible marker for this was not observed in their experiments. An in vitro study likewise found that the expression of the gene *NQO1* was altered after the treatment of human liver cells with 1,2,4-trichlorobenzene, which suggests that ROS are formed (US EPA 2020 b). However, quinones cause cytotoxicity even without ROS-mediated effects (see above) and may be able to initiate proliferation in the liver. Although one study reported negative findings with respect to possible tumour-promoting effects induced by the trichlorobenzenes, this study is not suitable for ruling out any such effects because the substance was administered by intraperitoneal injection in only 2 doses (see Section 5.7.1). Hexachlorobenzene is structurally similar to the trichlorobenzenes, but has a longer half-life in the body because it is highly chlorinated. For this reason, exposure to hexachlorobenzene caused similar, but much stronger effects (disruption of porphyrin metabolism, liver toxicity, development of liver tumours) in the animal model than 1,2,4-trichlorobenzene. Although it has yet to be fully explained, a complex carcinogenic mechanism of action has likewise been proposed for hexachlorobenzene. This involves the induction of CYPs, effects on iron homeostasis, disrupted porphyrin metabolism and indications for the activation of the Ah receptor (Greim 2002 a, available in German only; IARC 2001). The trichlorobenzenes may have a comparable mechanism of action in this respect as well: in addition to in vivo evidence of the induction of CYPs, in vitro and animal studies of 1,2,4-trichlorobenzene revealed effects on iron homeostasis and metabolism. These effects were caused by the induction of haem oxygenase or the formation of a methyl sulfone metabolite that induces δ-aminolaevulinate synthetase, leading to the disruption of porphyrin metabolism. In vivo findings for 2,3,7,8-tetrachlorodibenzo-*p*-dioxin demonstrated that interaction with the Ah receptor may be associated with porphyrin metabolism (increased formation of uroporphyrin) (Davies et al. 2008).

Tumour promotion may be caused also by the cytotoxicity of a substance (Holsapple et al. 2006). As explained at the beginning, reactive intermediates such as arene oxides and quinones may form during the metabolism of trichlorobenzene. These kinds of substances cause damage to the cells through binding (Bolton et al. 2000). Evidence for cytotoxicity was found in mammalian cells, but not for genotoxicity (see Section 5.6 and Section 5.8). The findings in the kidneys of male rats support the assumption that the adverse effects induced by the trichlorobenzenes are caused by their cytotoxicity. A possible explanation for this is α2u-globulin-mediated nephropathy which is caused by cytotoxicity (Laube et al. 2019) and is not relevant for humans because α2u-globulin does not occur in humans (den Besten et al. 1994).

In addition, in vitro evidence suggests that 1,2,4-trichlorobenzene interacts with the RXR receptor (US EPA 2020 b). In rodents, this form of interaction with receptors leads to cell hypertrophy in the liver, which, in turn, may precede tumour development. However, mechanistically, this is not relevant for humans (Hall et al. 2012; Lake et al. 2015). 1,2,3-Trichlorobenzene uncoupled oxidative phosphorylation of the respiratory chain in vitro, an effect that may occur also after exposure to the other 2 isomers. This uncoupling was accompanied by the release of potassium from the mitochondria in the rat liver and the inhibition of respiratory control (Ogata et al. 1981). Recent studies found an association between tumour promotion in the liver and interference in oxidative phosphorylation (Ashton et al. 2018; Santacatterina et al. 2016).

Overall, based on the available data, including the negative findings for genotoxicity, the high spontaneous incidence of liver tumours in B6C3F1 mice and the lack of statistical significance for the tumour findings in rats (see Section 5.7), it can be concluded that the liver tumours were induced by the tumour-promoting effects of the trichlorobenzenes. The organ toxicity observed in the liver and kidneys was caused, among other factors, by cytotoxic intermediates and subsequent effects such as the induction of enzymes. These are also responsible for the most sensitive end point, the disruption of porphyrin metabolism in rats. The irritation induced by the trichlorobenzenes in animal studies was probably caused by the degreasing effects of the substances.

## 3 Toxicokinetics and Metabolism

In the documentation published in 1990 (Henschler 1992), the metabolism of the trichlorobenzenes was described in detail. The data for toxicokinetics and metabolism are briefly presented again below together with recent study findings.

### 3.1 Absorption, distribution and elimination

#### 3.1.1 Inhalation

There are no data available for exposure by inhalation; however, it can be concluded that the isomer 1,2,4-trichlorobenzene is taken up by inhalation because adverse, substance-induced effects occurred in inhalation studies with animals (Coate et al. 1977; Dow Chemical Company 1977; Kociba et al. 1981).

#### 3.1.2 Oral administration

Within 24 hours of being given ^14^C-labelled 1,2,4-trichlorobenzene orally at 10 mg/kg body weight, female rhesus monkeys had excreted 40% of the applied dose with the urine, but only about 1% with the faeces. During the same time period, male albino rats that were treated with the same dose excreted 84% and 11% of the applied dose with urine and faeces, respectively (ATSDR 2014; Henschler 1992).

Five days after Chinchilla rabbits were given 1,2,3-trichlorobenzene, 1,2,4-trichlorobenzene and 1,3,5-trichlorobenzene (500 mg/kg body weight in each case) by gavage, the animals had excreted 78%, 42% and 32%, respectively, of the applied dose in the form of trichlorophenol or conjugates (Jondorf et al. 1955). In Chinchilla rabbits given an oral dose of 1,3,5-trichlorobenzene of 500 mg/kg body weight, extensive distribution throughout the body was observed: 8 days after administration, 23%, 22% and 13% of the applied dose was recovered in the intestines, carcass and faeces, respectively, in addition to 5% in the body fat and 5% in the fur (Parke and Williams 1960).

Sprague Dawley rats were given oral doses of ^14^C-labelled 1,2,4-trichlorobenzene of 181.5 mg/kg body weight daily on 7 consecutive days and then observed for 16 days. Initially, the highest level of radioactivity was found in the adrenal glands. Radioactivity was no longer detectable in the adrenal glands 11 days after administration. A high level of radioactivity (2033 dpm/g) was found in the abdominal fat on the first day after day 7 of administration. The amount of radioactivity had decreased to about 400 dpm/g 16 days after administration. In the liver, the corresponding levels of radioactivity were 1075 dpm/g and 317 dpm/g, respectively. The amount excreted with the urine was equivalent to 72% of the applied dose; radioactivity continued to be detected for 14 days after the last administration. After day 15, radioactivity was no longer detected in the faeces. Overall, 4% of the applied dose was excreted via this route (Smith and Carlson 1980).

Seven groups of 4 male Charles River rats were given 10 mg of ^14^C-labelled 1,2,4-trichlorobenzene per gavage. One of the treatment groups was sacrificed and examined after 3, 6, 12, 24, 48, 72 and 96 hours. Urine and faeces samples were collected and analysed every 24 hours. The highest tissue concentration was detected after 3 hours in the fat. After 24 hours, 3.5% of the applied radioactivity was still present in the digestive tract with only 1% remaining in the other tissues. Within 24 hours, about 80% to 90% of the applied radioactivity was excreted with the urine and about 10% to 20% with the faeces. In the same study, groups of 2 female rhesus monkeys were given a single oral dose of 1,2,4-trichlorobenzene of 10 mg/kg body weight. The animals were examined after 1, 2, 4, 6, 8, 12, 24, 48, 72 and 96 hours. The amount excreted with the urine was equivalent to 56% to 73% of the applied dose. The distribution in the tissues was similar to that in rats; the highest levels were determined in the adipose tissue. After oral administration, less than 4% was excreted with the faeces, whereas 36% to 40% was recovered in the urine after 24 hours and 56% to 73% in the 4 days following dose administration (Smith et al. 1985).

In another study, 5 male Wistar rats were given an oral dose of ^14^C-1,2,4-trichlorobenzene of 50 mg/kg body weight. In this study, high levels of radioactivity were found in the body fat (81.33% and 57.15%, respectively) and skin (15% and 14%, respectively) 12 and 24 hours after administration. After 12 and 24 hours, the percentage of radioactivity recovered in the muscles accounted for 8.29% and 4.12%, in the small intestines 4.66% and 3.69% and in the liver 1.98% and 2.08%, respectively, of the applied dose. Over the course of 7 days, the amount of radioactivity in the tissues decreased and became evenly distributed. Within 7 days, the rats had excreted 83% of the applied dose (66% and 17% with the urine and faeces, respectively). Only 2.1% was eliminated with the exhaled air. In general, the largest fraction was elim­inated 3 days after dose administration. In the same study, 45% of the applied radioactivity was recovered in the bile. The authors suggested that the differences in excretion via the bile and faeces were due to enterohepatic circulation (Tanaka et al. 1986).

Groups of 5 male Sprague Dawley rats (SD rats) were given gavage doses of ^14^C-labelled 1,2,3-trichlorobenzene, 1,2,4-trichlorobenzene or 1,3,5-trichlorobenzene of 10 mg/kg body weight. For all 3 isomers, radioactivity was detected in the blood and tissues only 30 minutes after substance administration. After 24 hours, the highest concentrations of ^14^C-1,2,3-trichlorobenzene were found in the digestive tract, body fat, liver, kidneys and bladder. Radioactivity was no longer detectable in the brain, muscles, testes and seminal vesicles 7 days after dose administration. In the case of ^14^C-1,2,4-trichlorobenzene, the highest concentrations in the tissues were found in the fat, skin and muscles 12 hours after dose administration. Seven days later, radioactivity was no longer detectable in the brain, spleen, muscles, testes, seminal vesicles or the prostate gland. In all examined tissues except for the adrenal glands, radioactivity was no longer present in measurable amounts beyond the naturally occurring background radiation 28 days after dose administration. In the case of ^14^C-1,3,5-trichlorobenzene, the highest concentrations in the tissues were found 24 hours after application. The highest levels were determined in the body fat with smaller fractions found in the digestive tract, salivary glands, kidneys, adrenal glands and bladder, liver, pancreas, epidermis, prostate gland, skin and lungs. The lowest concentrations were found in the seminal vesicles. Although the radioactivity in the individual organs decreased steadily until day 7 following treatment with ^14^C-1,2,3-trichlorobenzene and ^14^C-1,2,4-trichlorobenzene, reaching the limit of detection, higher levels were found in all tissues after administration of ^14^C-1,3,5-trichlorobenzene and significant levels of radioactivity were still detectable on days 28 and 56. For all isomers, the data show that most of the radioactivity in the skin, liver and body fat can be traced back to the parent substances, whereas most of the radioactivity in the muscles and kidneys can be attributed to metabolites with higher polarity. Within 24 hours, 92% of the applied 1,2,3-isomer was excreted with the urine and faeces. Within 48 hours, the total excreted amount increased to 95% of the applied dose. After 24 and 48 hours, respectively, 56% and 59% of the excreted radioactivity was recovered in the urine. Similarly high levels were determined for the excretion of 1,3,5-trichlorobenzene: 82% and 89% of the applied dose was found in the urine and the faeces after 24 and 48 hours, respectively. The amount recovered in the urine after 24 and 48 hours was equivalent to 47% and 50% of the applied radioactivity, respectively. The elimination kinetics were explained using a two-compartment model: in the first compartment, the half-lives for 1,2,3-trichlorobenzene, 1,2,4-trichlorobenzene and 1,3,5-trichlorobenzene were determined to be 9, 12 and 8 hours, respectively, and in the second compartment, the terminal half-lives were 145, 93 and 68 hours, respectively. However, the reliability of the calculations is regarded as uncertain because the fractions of parent substance and metabolites in the excreted radio­activity were not determined at each reading (Chu et al. 1987).

The finding of Chu et al. (1987) that 1,3,5-trichlorobenzene accumulates in greater amounts in the tissues of rats than the other isomers was confirmed in another study. In this study, 130 male and 130 female Sprague Dawley rats were divided into groups of 10 animals and given different amounts of ^14^C-labelled 1,3,5-trichlorobenzene, 1,2,4-trichlorobenzene or 1,2,3-trichlorobenzene with the feed (0, 10, 100 or 1000 mg/kg) for 13 weeks. In the high dose group, 1,3,5-trichlorobenzene accumulated in greater amounts than 1,2,4-trichlorobenzene or 1,2,3-trichlorobenzene. After administration of 1,3,5-trichlorobenzene, the concentrations in the body fat were about 76 mg/kg in the male animals and about 49 mg/kg in the females. These, in turn, were much higher than the concentrations in the liver: about 4.3 mg/kg in the males and about 1.9 mg/kg in the females. In general, the following order was established for the dose-dependent accumulation of the isomers in the body fat: 1,3,5-trichlorobenzene > 1,2,4-trichlorobenzene > 1,2,3-trichlorobenzene (Côté et al. 1988).

Within 24 hours after 5 mg of ^14^C-labelled 1,2,4-trichlorobenzene were given orally to Sprague Dawley rats fitted with bile duct cannulas, more than 60% of the applied dose was excreted with the bile, 21% with the urine and 2% with the faeces. Animals without bile duct catheters excreted 70% and 9%, respectively, of the applied radioactivity with the urine and faeces within 24 hours. This shows that the substance undergoes enterohepatic circulation (Bakke et al. 1992).

#### 3.1.3 Dermal application

No quantitative data are available for the absorption of trichlorobenzene through the skin. Animal studies found evidence of systemic toxicity after dermal application of 1,2,4-trichlorobenzene or a technical mixture of trichlorobenzenes containing 1,2,4-trichlorobenzene (ATSDR 2014; Brown et al. 1994; Henschler 1992). Due to their similar physicochemical properties (including molar mass, log K_OW_, water solubility), the 3 trichlorobenzene isomers can be assumed to behave in a similar manner with respect to absorption through the skin. Theoretical models predict that the compounds are absorbed through the skin. By applying the models of Fiserova-Bergerova et al. (1990) and IH SkinPerm (Tibaldi et al. 2014), fluxes of 287.1 and 6.35 µg/cm^2^ and hour, respectively, were calculated for a saturated aqueous solution of the isomer that has been found to penetrate most readily, namely 1,2,4-trichlorobenzene. Assuming the exposure of 2000 cm^2^ of skin for 1 hour, this would result in the absorption of 574.2 and 12.7 mg, respectively, of 1,2,4-trichlorobenzene.

#### 3.1.4 Intravenous injection

After female rhesus monkeys and male albino rats were given intravenous injections of 10 mg of ^14^C-labelled 1,2,4-trichlorobenzene, within 24 hours of treatment the monkeys excreted 22% and 0%, respectively, and the rats 78% and 7%, respectively, with the urine and faeces (ATSDR 2014; Henschler 1992).

Seven groups of 4 male Charles River rats were given 10 mg of ^14^C-labelled 1,2,4-trichlorobenzene by intravenous injection. One group of animals was sacrificed and examined after 3, 6, 12, 24, 48, 72 and 96 hours. Within 48 hours, 83% to 86% of the applied dose was excreted with the urine and 12% with the faeces. After 6 hours, the highest levels of radioactivity were found in the blood plasma, liver and kidneys. The amount of radioactivity in the adipose tissue was higher and persisted there longer than in any of the other examined tissues (liver, kidneys). Therefore, the radioactivity was still detected in the fat 96 hours later. After 12 hours, the highest level of radioactivity was found in the intestinal tissue, which was interpreted by the authors as evidence of enterohepatic circulation. In the same study, groups of 2 female rhesus monkeys were given a single 1,2,4-trichlorobenzene dose of 10 mg/kg body weight by intravenous injection. The animals were examined after 1, 2, 4, 6, 8, 12, 24, 48, 72 and 96 hours. About 38% of the applied dose was excreted with the urine; 22% of the excreted amount was recovered within the first 24 hours. Again, distribution in the tissues was highest in the adipose tissue and comparable to the distribution found in rats. Thus, smaller amounts of radioactivity were excreted after intravenous injection and at a slower rate than after oral administration (see Section 3.1.2). The explanation given was that after intravenous administration, the lipophilic substance is preferentially distributed in the adipose tissue, whereas after oral administration, the substance is converted to polar metabolites in the liver. Less than 4% was excreted with the faeces after intravenous administration (Smith et al. 1985).

#### 3.1.5 Intraperitoneal administration

Groups of 3 to 4 male Wistar rats were given a single dose of 1,2,4-trichlorobenzene of 250 mg/kg body weight (equivalent to 1.36 mmol/kg body weight) by intraperitoneal injection. The half-lives of 1,2,4-trichlorobenzene in the blood, liver and kidneys were 5.8, 5.2 and 6.2 hours, respectively. Larger amounts of 1,2,4-trichlorobenzene were found in the adipose tissue than in the blood, liver or kidneys (Kato et al. 1993).

#### 3.1.6 Summary

Overall, the findings in animal models show that the trichlorobenzenes are rapidly absorbed and then distributed systemically. However, it should be taken into consideration that the use of lipophilic vehicles such as corn oil may increase the absorption of oral doses because the trichlorobenzenes themselves are lipophilic. This has been demonstrated in the past for hexachlorobenzene (Koss and Koransky 1975). Tissues with the highest concentrations are the body fat, skin, liver, kidneys, bladder and the digestive tract. The following order was determined for the 3 isomers for the amount of isomer that accumulates in the body fat and liver: 1,3,5-trichlorobenzene > 1,2,4-trichlorobenzene > 1,2,3-trichlorobenzene. On average, the amount in adipose tissue was 10 times higher than the amount found in the liver. Within 24 hours of oral administration, 50% to 80% of the applied radioactivity was excreted with the urine. This has been identified as the primary excretory pathway for all isomers. Excretion with the faeces plays a subordinate role. The elimination kinetics after oral administration were described using a two-compartment model. Although the method used to collect the half-life data was not reliable, the study determined half-lives for 1,2,3-trichlorobenzene, 1,2,4-trichlorobenzene and 1,3,5-trichlorobenzene of 9, 12 and 8 hours, respectively, in the first compartment and of 145, 93 and 68 hours, respectively, in the second compartment. The half-lives for 1,2,4-trichlorobenzene in the blood, liver and kidneys of rats after intraperitoneal administration were 5.8, 5.2 and 6.2 hours, respectively. Studies of the 1,2,4-isomer found evidence of enterohepatic circulation.

### 3.2 Metabolism

As a supplement to the diagrams published in the documentation from 1990 (Henschler 1992), Figure 1, Figure 2 and Figure 3 show the metabolic pathways of the trichlorobenzenes.

#### 3.2.1 In vitro studies

In vitro investigations with human microsomes found that of the enzymes involved in oxidative metabolism (CYP1A1, CYP1A2, CYP3A4, CYP2E1 and CYP2D6), the enzyme CYP2E1 possesses the highest metabolic activity towards 1,2,4-trichlorobenzene. In this study, after incubation with microsomes from 22 human livers, 1,2,4-trichlorobenzene was metabolized mainly by CYP2E1 to 2,4,5-trichlorophenol, 2,3,5-trichlorophenol and 2,3,4-trichlorophenol via arene oxides as intermediates. The formation of 2,3,6-trichlorophenol was attributed to the involvement of CYP3A4 (Bogaards et al. 1995).

1,2,4-Trichlorobenzene was incubated with the liver microsomes of male rats pre-treated with dexamethasone. Not only various trichlorophenols, but also trichlorohydroquinones formed. The trichlorohydroquinones bound to microsomal proteins; the metabolism as well as the binding of the metabolites to proteins was catalysed by CYP. Consequently, these enzymatic reactions were inhibited by the removal of NADPH and the addition of the inhibitor metyrapone. Furthermore, protein binding was almost completely blocked by the addition of glutathione, and water-soluble metabolites were formed. Mainly 2,3,6-trichlorophenol formed as a result of induction with dexamethasone. Without pre-treatment with dexamethasone, mainly 2,4,5-trichlorophenol formed. 2,4,6-Trichlorophenol formed to a lesser extent, and only trace amounts of 2,3,4-trichlorophenol and 2,3,5-trichlorophenol were found (den Besten et al. 1991 a). The formation of quinones was attributed to the further oxidation of the phenolic intermediates (den Besten et al. 1991 a, b).

#### 3.2.2 In vivo studies

The data from animal studies that are included here are based on studies of the metabolism after oral, intraperitoneal and intravenous administration. For all of the isomers, it is plausible that intermediates (arene oxides) are formed by oxidation that are then converted to phenolic metabolites and conjugated.

In Chinchilla rabbits treated with the 3 trichlorobenzene isomers in single oral doses of 500 mg/kg body weight, the metabolites were found to be mainly 2,3,4-trichlorophenol in addition to 3,4,5-trichlorophenol and 3,4,5-trichloro­catechol. After 5 days, 62% of the applied radioactivity was recovered in the urine in the form of conjugated metabolites and 4% as free trichlorophenols. 1,2,4-Trichlorobenzene was converted mainly to 2,4,5-trichlorophenol and 2,3,5-trichloro­phenol; 38% of the dose was present in the form of conjugates and 1.5% as free trichlorophenols. According to this study, 1,3,5-trichlorobenzene was metabolized only to 2,4,6-trichlorophenol and only 23% of the dose was found in the urine in conjugated form and 0.5% as free trichlorophenol (Jondorf et al. 1955). In another study of 1,3,5-trichlorobenzene, 2,4,6-trichlorophenol was recovered in the urine of rabbits in the first 4 days after administration (500 mg/kg body weight by gavage). By day 9 after treatment, also another metabolite, 4-chlorophenol, was found in the urine (Parke and Williams 1960).

In male rabbits treated intraperitoneally with a single dose of 1,2,3-trichlorobenzene of 300 mg/kg body weight, the following metabolites were identified in the urine: 2,3,4-trichlorophenol, 2,3,6-trichlorophenol and 3,4,5-trichlorophenol. Following administration of 1,2,4-trichlorobenzene at the same dose, 2,3,5-trichlorophenol and 2,4,5-trichlorophenol were detected in the urine as metabolites. 2,3,5-Trichlorophenol and 2,4,6-trichlorophenol were found after administration of 1,3,5-trichlorobenzene. About 4 times more 2,3,5-trichlorophenol formed from 1,2,4-trichlorobenzene than from 1,3,5-trichlorobenzene (Kohli et al. 1976). All metabolites were separated by chromatography and identified by mass spectrometry. As the urine was acidified during extraction, the metabolites that were detected may have been excreted as conjugates.

The metabolism was found to differ by species after both oral and intravenous administration of ^14^C-labelled 1,2,4-trichlorobenzene to female rhesus monkeys and male albino rats at a dose of 10 mg/kg body weight. Up to 24 hours after administration, the main urinary metabolites found in monkeys were the 2 isomers of 3,4,6-trichloro-3,5-­cyclohexadiene-1,2-diol glucuronide (48% to 61% of the radioactivity excreted with the urine) in addition to 2,4,5-trichlorophenol glucuronide and 2,3,5-trichlorophenol glucuronide (14% to 37% of the radioactivity excreted with the urine) and free trichlorophenols (1% to 37% of the radioactivity excreted with the urine). In rats, the main urinary metabolites after both oral and intravenous administration were the 2,4,5-isomer and the 2,3,5-isomer of *N*-acetyl-*S*-(trichlorophenyl)-L-cysteine (60% to 62% of the radioactivity excreted with the urine). 2,4,5-Trichlorothiophenol and 2,3,5-trichloro­thiophenol (about 28% of the radioactivity excreted with the urine after intravenous administration and 33% after oral administration) in addition to 2,4,5-trichlorophenol and 2,3,5-trichlorophenol (10% intravenous, 1% oral) were likewise detected in the urine. On this basis, it is concluded that glutathione in rats and glucuronic acid in rhesus monkeys are the main conjugation partners of the metabolites (ATSDR 2014; Henschler 1992). After conjugation with glutathione, the substances are further broken down into thioethers and converted to methyl sulfoxides and methyl sulfones.

After rats were given a single oral dose of 1,2,4-trichlorobenzene of 50 mg/kg body weight, mainly conjugated metabolites (90%) were excreted with the urine. Free and conjugated 2,4,5-trichlorophenol and 2,3,5-trichlorophenol were found together with small amounts of 5-sulfhydryl and 6-sulfhydryl, methylthio, methylsulfoxide and methylsulfone derivatives of 1,2,4-trichlorobenzene. Small amounts of unchanged 1,2,4-trichlorobenzene and of *o*-dichlorobenzene, *m*-dichlorobenzene and *p*-dichlorobenzene (probably dechlorinated by intestinal bacteria) were found in the exhaled air. Unchanged 1,2,4-trichlorobenzene was detected in the faeces (Tanaka et al. 1986). In vitro studies already demonstrated that intestinal bacteria degrade 1,2,4-trichlorobenzene to dichlorobenzenes and monochlorobenzenes (Tsuchiya and Yamaha 1984).

After intraperitoneal administration of 1,3,5-trichlorobenzene to mice, dichlorinated mercapto, methylthio, methyl­sulfinyl and methylsulfonyl metabolites were found in adipose tissue and mercapto, methylsulfide, methylsulfoxide, methylsulfone and *o*-hydroxymercapto, *o*-hydroxymethylsulfide, *o*-hydroxysulfoxide and *o*-hydroxysulfone metabolites were recovered in the urine. The postulated mechanism of dechlorination is the replacement of a chlorine atom by sulfur (Mio and Sumino 1985).

The following metabolites were identified in the excretory products of Sprague Dawley rats fitted with bile duct catheters after exposure to 5 mg of 1,2,4-trichlorobenzene: *S*-(trichlorophenyl)-*N*-(acetyl)cysteine was the main metabolite, while *S*-(dichlorohydroxyphenol)-*N*-(acetyl)cysteine, likewise a mercapturic acid, trichlorothiophenol and trichlorophenol were found in smaller amounts. The occurrence of these metabolites is likewise regarded as evidence of glutathione conjugation that was preceded by the formation of phenolic metabolites. The metabolite trichlorothiophenol was found in the bile in larger amounts than in the urine. Trichlorothiophenols were probably formed by the intestinal flora during enterohepatic circulation (Bakke et al. 1992).

This assumption was confirmed by a later study that found that 2,3,5-trichlorophenyl methyl sulfone was formed in much smaller amounts by rats pre-treated with an antibiotic. In the study, groups of 3 to 4 male Wistar rats were given an oral dose of 1,2,4-trichlorobenzene of 400 mg/kg body weight daily on 5 consecutive days. The following metabolites were detected and identified in alkalinized urine samples: 2,3,5-trichlorophenyl methyl sulfide and 2,4,5-trichloro­phenyl methyl sulfide, 2,3,5-trichlorophenyl methyl sulfoxide and 2,4,5-trichlorophenyl methyl sulfoxide in addition to 2,3,5-trichlorophenyl methyl sulfone and 2,4,5-trichlorophenyl methyl sulfone. Other groups of animals were given the substance by intraperitoneal injection in a single dose of 250 mg/kg body weight (equivalent to 1.36 mmol/kg body weight). Smaller amounts of the metabolites 2,3,5-trichlorophenyl methyl sulfone and 2,4,5-trichlorophenyl methyl sulfone were detected in the body fat, liver, kidneys and blood; 2,3,5-trichlorophenyl methyl sulfone was still detectable in these organs even 120 hours after administration. The explanation given for this was the strong lipophilicity of the metabolite and its continuous formation as a product of the metabolism of the 1,2,4-trichlorobenzene remaining in the body fat. This is evidence that, in the case of the 1,2,4-isomer, glutathione conjugation may be followed by further degradation to thioethers and conversion to methyl sulfoxides and methyl sulfones. The process involves the gradual degradation of the glutathionyl residue via cleavage of glutamate, glycine or cysteine while retaining the thiol group and *S*-methylation followed by the oxidation of sulfur (Kato et al. 1993).

In studies with Wistar rats, the animals were either pre-treated with buthionine sulfoximine (2 subcutaneous injections in saline solution at an interval of 6 hours) or were not pre-treated. The animals were then given 1,2,4-trichlorobenzene or the metabolites 2,3,5-trichlorophenyl methyl sulfone and 2,4,5-trichlorophenyl methyl sulfone by injection. The pre-treated rats were found to have lower concentrations of methyl sulfone metabolites in the liver than the animals that had not been pre-treated. As pre-treatment with buthionine sulfoximine decreases the glutathione levels in the liver, these results support the hypothesis that methyl sulfone metabolites are formed from the trichlorobenzenes after earlier conjugation with glutathione. The studies additionally found that haem oxygenase, but not δ-aminolaevulinate synthetase, was induced by 1,2,4-trichlorobenzene in the pre-treated animals, whereas δ-aminolaevulinate synthetase was induced after administration of 2,3,5-trichlorophenyl methyl sulfone. It can therefore be concluded that 2,3,5-trichlorophenyl methyl sulfone plays an important role in the induction of δ-aminolaevulinate synthetase, whereas haem oxygenase is activated mainly by 1,2,4-trichlorobenzene (Kato and Kimura 2002).

#### 3.2.3 Summary

The 1,2,4-isomer is oxidized mainly by CYP2E1. All 3 trichlorobenzene isomers initially form arene oxides followed by polar phenolic intermediates. In phase II of metabolism, the phenolic intermediates, which vary depending on the metabolized isomer, undergo species-dependent conjugation with glutathione, glucuronic acid or sulfate. In rats treated with the 1,2,4-isomer, glutathione conjugation may be followed by further degradation to thioethers and by conversion to methyl sulfoxides and methyl sulfones (2,3,5-trichlorophenyl methyl sulfone and 2,4,5-trichlorophenyl methyl sulfone). There is no evidence that 2,3,5-trichlorophenyl methyl sulfone, the metabolite that is assumed to play a critical role, forms from 1,2,3-trichlorobenzene and 1,3,5-trichlorobenzene. In the metabolism of mice, 1,3,5-trichlorobenzene is likewise broken down to sulfur-containing metabolites such as the methyl sulfones; however, glutathione conjugation at a chlorine position leads to dechlorination, forming dichloro compounds.

**Fig.1:**
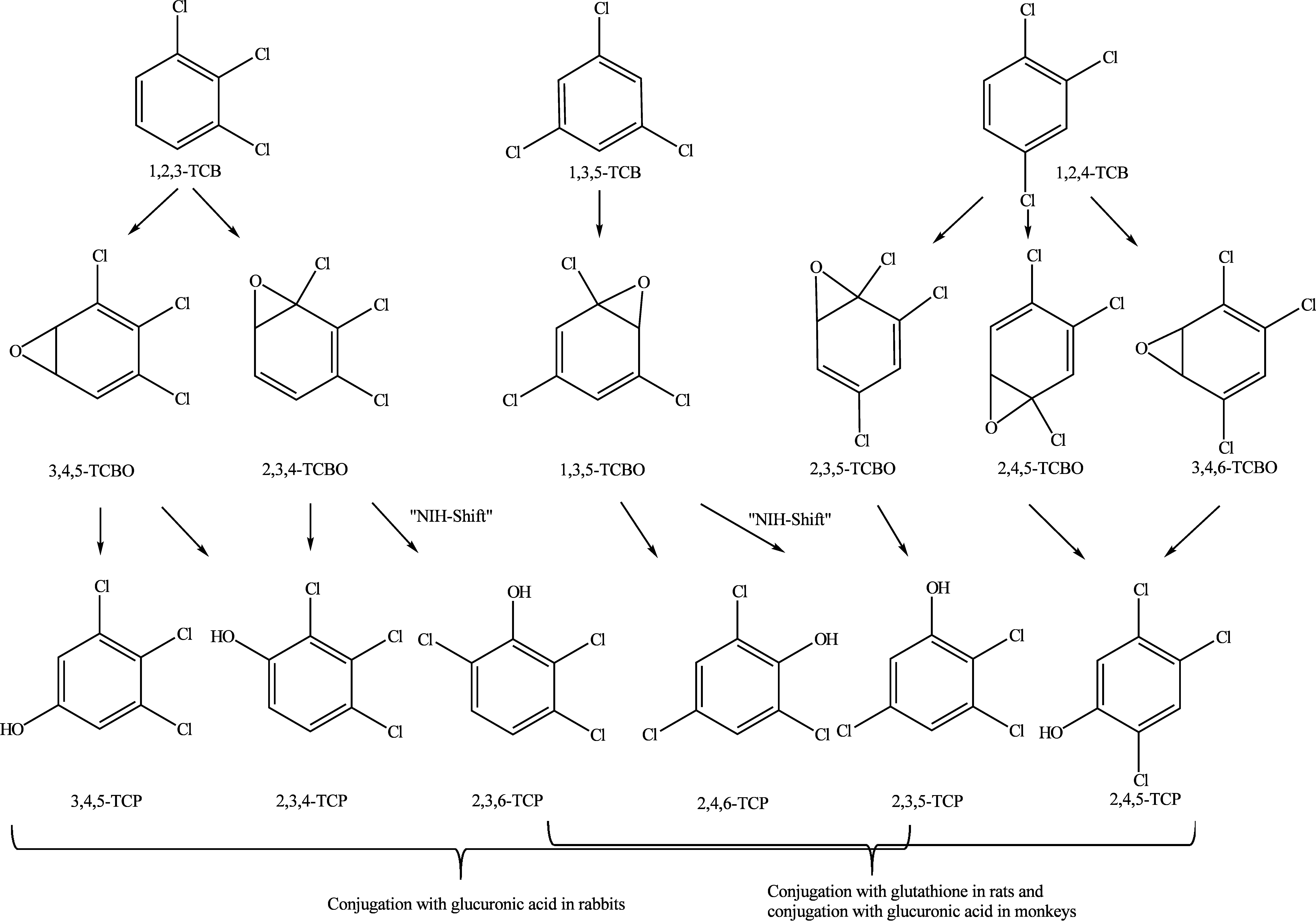
Postulated metabolic pathways for 1,2,3-trichlorobenzene, 1,2,4-trichlorobenzene and 1,3,5-trichlorobenzene (according to ATSDR (2014))

TCB: trichlorobenzene; TCBO: trichlorobenzene epoxide; TCP: trichlorophenol

**Fig.2:**
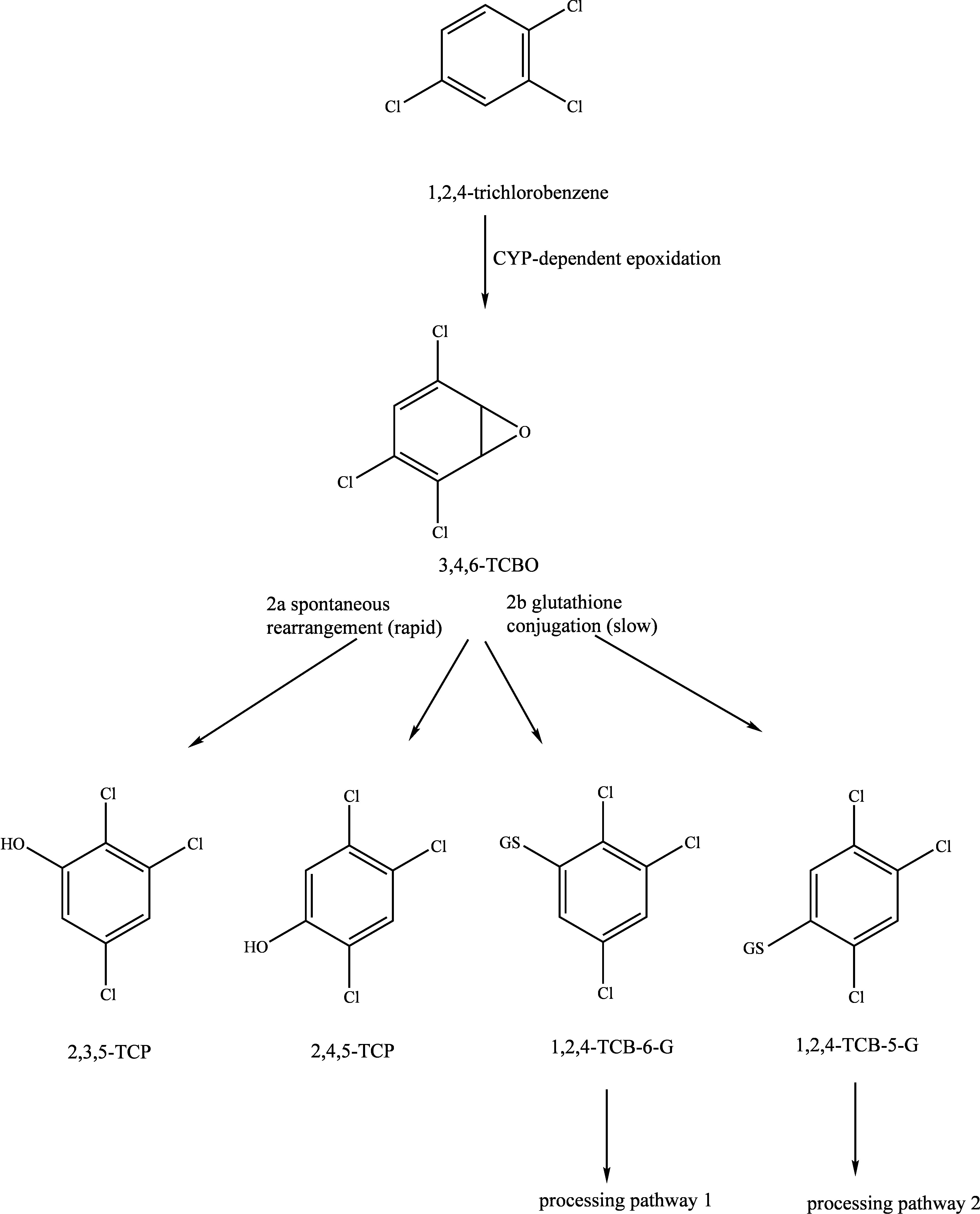
Postulated metabolic pathway for 1,2,4-trichlorobenzene after intraperitoneal administration to rats (according to Kato et al. (1993) and Bakke et al. (1992))

TCBO: trichlorobenzene epoxide; TCP: trichlorophenol; TCB-G: trichlorobenzene glutathione adduct

**Fig.3:**
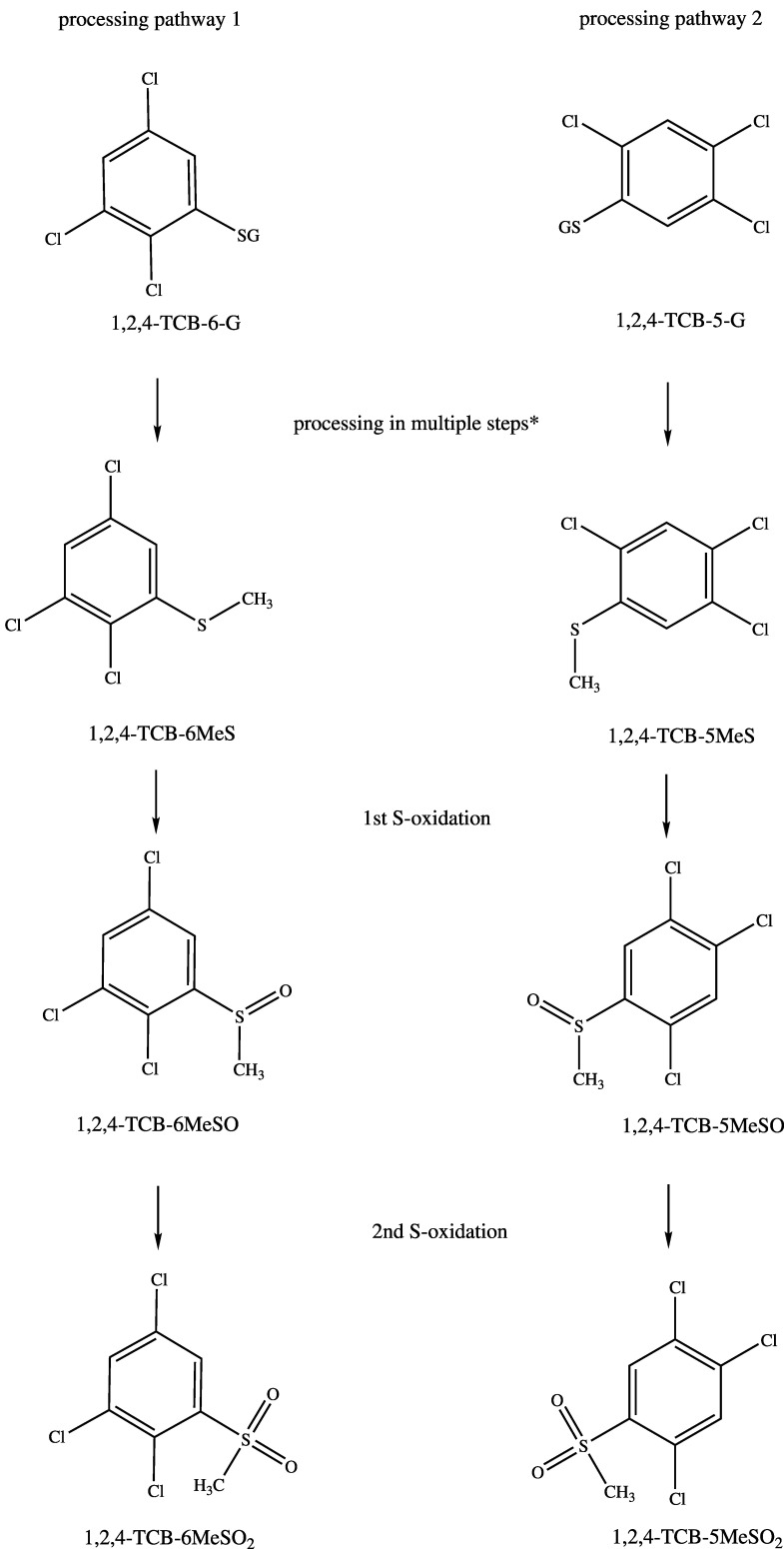
Postulated metabolic pathway for the processing of 1,2,4-trichlorobenzene after conjugation with glutathione in rats (according to Kato et al. (1993) and Bakke et al. (1992))

*Multi-step processing: gradual functional degradation of glutathione via

a) glutamate cleavage,
b) glycine cleavage,
c) cysteine cleavage while retaining the thiol group,
d) *S*-methylation

TCB: trichlorobenzene; TCB-G: trichlorobenzene glutathione adduct; TCB-MeS: trichlorophenyl methyl sulfide; TCB-MeSO: trichlorophenyl methyl sulfoxide; TCB-MeSO2: trichlorophenyl methyl sulfone

## 4 Effects in Humans

The odour threshold for 1,2,4-trichlorobenzene is between 1.4 and 3 ml/m^3^ (EC 2003). Unpublished reports and a personal communication described slight irritation of the eyes and respiratory tract in industrial workers who were exposed to 1,2,4-trichlorobenzene concentrations of 3 to 5 ml/m^3^ (23 to 37 mg 1,2,4-trichlorobenzene/m^3^) (EC 2003; Henschler 1992). Adverse effects on the blood after inhalation exposure to trichlorobenzene (isomer not specified) were described in the documentation published in 1990 (Henschler 1992). In addition, severe haemoptysis developed in an adult male who had inhaled trichlorobenzene (no other details) for several hours while carrying out repair work. Production workers developed chloracne after exposure to trichlorobenzene (no other details) (ATSDR 2014). None of the studies or accident reports mentioned above include data that can be used to characterize the isomer or determine the exposure concentrations, or that provide detailed information about the exposed persons. This strongly limits the relevance of the reports for the evaluation.

Exposure to hexachlorobenzene gave rise to adverse effects in humans in the form of marked liver toxicity and porphyria (Peters et al. 1987). In animal studies, hexachlorobenzene was found to disrupt porphyrin metabolism (Greim 2002 a). This effect was likewise observed in rats after exposure to trichlorobenzenes. Therefore, these types of effects are likely to occur in humans after exposure to the trichlorobenzenes at high doses (ATSDR 2014). However, there are no known studies of these effects.

## 5 Animal Experiments and in vitro Studies

### 5.1 Acute toxicity

The documentation published in 1990 included information on acute toxicity and the LD_50_ values for various animal species. Overall, the data show that 1,2,4-trichlorobenzene has higher toxic potency than 1,2,3-trichlorobenzene and 1,3,5-trichlorobenzene and that the acute toxic effects induced by all trichlorobenzenes affect mainly the liver and kidneys in addition to the adrenal glands and mucous membranes (Henschler 1992). The LD_50_ for 1,2,3-trichlorobenzene after oral exposure of rats was 1830 mg/kg body weight and the LD_50_ in mice after intraperitoneal injection was 1390 mg/kg body weight. The oral LD_50_ for the 1,2,4-isomer was in the range of 756 to 880 mg/kg body weight in rats and 300 to 766 mg/kg body weight in mice. The LD_50_ in mice after intraperitoneal injection was 1223 mg/kg body weight and the LD_50_ in rats after percutaneous treatment was 6139 mg/kg body weight. The LD_50_ values after oral exposure to 1,3,5-trichlorobenzene were in the range of 1800 to 2800 mg/kg body weight for rats and 3550 to 3402 mg/kg body weight for mice. A value of 2260 mg/kg body weight was determined for mice after intraperitoneal injection (Henschler 1992). Acute toxicity studies which used solubilizers such as Lutrol or Chremophor as vehicles have not been included again in this addendum. An LD_50_ for 1,3,5-trichlorobenzene of 800 mg/kg body weight was determined in rats after oral exposure (NCBI 2020 c).

Rats (no other details) were exposed head-only for 1 hour to vapour saturated with a 1,3,5-trichlorobenzene concentration of 1209 ml/m^3^. None of the 8 male and 8 female rats died during exposure or during the 2-week observation period. The only findings that were considered substance-induced were slight irritation in the area around the eyes and reduced body weight gains during the observation period (Jorgenson et al. 1976).

Groups of 3 male Wistar rats aged 10 to 14 weeks were given a single intraperitoneal injection of 1,2,4-trichlorobenzene doses of 1, 2 or 4 mmol/kg body weight (dissolved in arachis oil). These are equivalent to 1,2,4-trichlorobenzene doses of 181.5, 362.9 and 725.8 mg/kg body weight, respectively. Control animals were given injections of pure arachis oil. At regular intervals, blood samples were analysed (24, 48 and 72 hours after administration, thyroxine, triiodothyronine, blood urea nitrogen and alanine aminotransferase activity), body weights were determined and unusual findings (daily) were recorded. The animals were sacrificed 72 hours after treatment and the livers and kidneys were weighed and examined histopathologically. Additional groups of 4 male rats were given a single 1,2,4-trichlorobenzene dose of 181.5 or 362.9 mg/kg body weight by intraperitoneal injection. These animals were sacrificed and examined only after 216 hours (9 days). During this period, data for blood parameters (every 24 hours) and body weights were recorded daily. The livers and kidneys were examined histopathologically. Additional groups of 3 male rats were given a single 1,2,4-trichlorobenzene dose of 0 or 725.8 mg/kg body weight by intraperitoneal injection. The animals and control rats were sacrificed 0 (controls only), 2, 5, 10, 24 or 48 hours after administration, their livers and kidneys were homogen­ized and the glutathione levels and blood parameters were determined. Three additional male rats per group were treated with a 1,2,4-trichlorobenzene dose of 0 or 181.5 mg/kg body weight and the glutathione levels in the livers and kidneys were determined 0 (controls only) and 8 hours after administration. All treated animals were found to have lower body weights and increased liver weights 72 hours after administration. This effect was still statistically signifi­cant 9 days after administration. The alanine aminotransferase activity in the plasma of the exposed rats increased with the dose. Two hours after injection, the glutathione levels in the livers of the exposed animals were reduced. This effect increased in severity up to 5 hours after administration. After 72 hours, centrilobular hypertrophy and cell degeneration, necrosis and cell proliferation were found in the liver tissues collected from the medium and high dose groups. Kidney damage (tubular degeneration, dilation) was observed after exposure to 1,2,4-trichlorobenzene at a dose of 725.8 mg/kg body weight and nephrosis after exposure to 362.9 mg/kg body weight. After 72 hours, protein droplets were detected in the epithelial cells of the proximal tubules. In addition, the thyroxine levels in the blood plasma of the exposed animals were decreased with statistical significance up to 24 hours after exposure. This effect was already noticeable in the group given 725.8 mg/kg body weight, the highest dose tested, after 5 hours (den Besten et al. 1991 b).

### 5.2 Subacute, subchronic and chronic toxicity

#### 5.2.1 Inhalation

The documentation from 1990 (Henschler 1992) included data for the effects caused by inhalation exposure. These studies are briefly presented again below together with additional study findings.

In a study, 4 male and 4 female Alderley Park rats were exposed to 1,2,4-trichlorobenzene in a concentration of 20 ml/m^3^ for 6 hours a day for 20 days, and 2 male and 2 female rats were exposed to the substance in concentrations of 70 or 200 ml/m^3^ for 15 days. The 1,2,4-trichlorobenzene that was used for the test was contaminated with up to 20% 1,2,3-trichlorobenzene. Lethargy, reduced body weight gains and lacrimation were induced by exposure to 70 or 200 ml/m^3^, but no histological changes were observed in the lungs, heart, intestines, adrenal glands, spleen and thymus gland (Gage 1970).

Cynomolgus monkeys (9 male animals per group), Sprague Dawley rats (30 male animals per group) and rabbits (New Zealand White rabbits, 16 male animals per group) were exposed to 1,2,4-trichlorobenzene concentrations of 0, 25 or 100 ml/m^3^ for 7 hours a day, on 5 days a week, for a period of 26 weeks. The purity of the test substance was 99%. Slight histopathological changes in the liver and kidneys of the rats were found after 4 and 13 weeks, respectively, but not after 26 weeks of exposure. Progressive hepatocytomegaly that increased in severity with the concentration was determined in all treated rats, but the effect did not intensify over time. In addition, vacuolation of the hepatocytes and an increased incidence of granulomas were observed in the animals after 4 weeks; these effects were not dependent on the concentration. In the kidneys of the rats, hyaline degeneration was observed in the inner cortex zone; however, the incidence was increased in the high concentration group only from week 13 onwards. Toxic effects were not noticeable in the other animal species that were examined. The histopathological examination did not find irritation in the respiratory tract in the 3 species of animal. The results of the lung function tests carried out with the treated monkeys were comparable to those of the control animals. Likewise, no toxic effects on behaviour were observed (Coate et al. 1977).

In an inhalation study, 20 male Sprague Dawley rats, 2 male beagle dogs and 4 male New Zealand White rabbits were exposed to 1,2,4-trichlorobenzene concentrations of 0, 30 or 100 ml/m^3^. The purity of the test substance was above 99%. The animals were exposed for 7 hours a day, on 5 days a week, for 44 days. The relative and absolute liver weights were increased in rats by about 11% and in dogs by about 27% to 30%; in both species, these effects were detected in the high concentration group. In the rats, the relative and absolute kidney weights were likewise increased by about 8% in this concentration group. The urine of the animals was examined after 15 and 30 days and increased levels of coproporphyrin and uroporphyrin were determined in rats exposed to 30 and 100 ml/m^3^. These effects did not increase in severity over time. They were attributed to the induction of enzymes in the liver. No further substance-related effects on other organs, including the respiratory tract and the nasal mucosa, and on haematological and biochemical parameters in rats, rabbits and dogs were observed (Kociba et al. 1981).

Another study was carried out to evaluate these findings. In 1990, the results of this study were not available in their entirety for evaluation by the Commission. In this study, groups of 10 male and 26 female Sprague Dawley rats were exposed to 1,2,4-trichlorobenzene concentrations of 0, 3 or 10 ml/m^3^ for 6 hours a day, on 5 days a week, for 3 months (65 to 66 exposures in total). The purity of the test substance was above 99%. Interim sacrifices of 4 to 5 female animals from each concentration group were carried out during the exposure period after 2 weeks and 1, 2 and 3 months, and 2 and 4 months after the end of exposure. The porphyrin levels in the liver were analysed. Urine was collected and analysed at the same intervals (coproporphyrin, uroporphyrin and creatinine levels). At the end of the study, all remaining animals were sacrificed and the brains, kidneys, livers and lungs were examined by gross pathology. At the end of exposure, the only substance-related effects were increasing levels of porphyrin in the urine of the animals of the 10 ml/m^3^ group; the increase was statistically significant. These effects were observed as early as after 2 weeks and did not become more severe during the 3 months of exposure. As the porphyrin levels in the urine of the animals were no longer increased 4 months after the end of exposure, this effect is regarded as reversible. At necropsy, no findings were detected in the organs of the animals that were examined. Unlike the data from earlier studies investigating inhalation and oral exposure to 1,2,4-trichlorobenzene, particularly the findings from the study of Kociba et al. (1981), the authors suggest that the disruption of porphyrin metabolism is both the earliest and the most sensitive effect in the sequence of adverse effects. This effect was investigated only in rats. The other toxic effects induced by 1,2,4-trichlorobenzene in the animal model (induction of liver enzymes, tissue damage, liver hypertrophy and increased liver weights) occurred only later or at higher concentrations. The authors derived a NOAEC (no observed adverse effect concentration) of 3 ml/m^3^ for the effects induced by 1,2,4-trichlorobenzene in the most sensitive species (rat). The LOAEC (lowest observed adverse effect concentration) for 1,2,4-trichlorobenzene was 10 ml/m^3^ (Dow Chemical Company 1977; US EPA 2009 b). The findings relating to the disruption of porphyrin metabolism are shown in Table 1.

**Tab.1:** Porphyrin excretion in the 24-hour urine of Sprague Dawley rats after inhalation exposure to 1,2,4-trichlorobenzene for 3 months (Dow Chemical Company 1977; US EPA 2009 b)

Parameter	Sex	Concentration [ml/m^3^]		
		0	3	10
Coproporphyrin excretion (µg)	♂	6.1 ± 3.4	8.5 ± 5.4	11.4 ± 5.9*
	♀	5.3 ± 3.4	2.4 ± 0.5	3.1 ± 1.0
Uroporphyrin excretion (µg)	♂	1.3 ± 1.0	2.2 ± 1.7	4.1 ± 2.4*
	♀	0.6 ± 0.3	0.7 ± 0.1	1.0 ± 0.2*
Coproporphyrin excretion (µg/mg creatinine)	♂	0.58 ± 0.33	0.78 ± 0.42	1.0 ± 0.5
	♀	0.67 ± 1.23	0.35 ± 0.14	0.32 ± 0.11
Uroporphyrin excretion (µg/mg creatinine)	♂	0.11 ± 0.05	0.17 ± 0.09	0.31 ± 0.12*
	♀	0.07 ± 0.04	0.10 ± 0.04	0.10 ± 0.02

*p < 0.05

In an inhalation study, 20 male and 20 female CD rats per concentration group were exposed to 1,3,5-trichlorobenzene concentrations of 0, 10, 100 and 1000 mg/m^3^ for 6 hours a day, on 5 days a week, for up to 15 weeks. The purity of the test substance was above 99%. The animals were examined after 4 weeks (5 per sex and concentration) and after 13 weeks of exposure (15 per sex and concentration). The histopathological examination of the groups exposed to 1,3,5-trichlorobenzene concentrations of 10 and 100 mg/m^3^, did not reveal adverse effects on the organs, including the respiratory tract. In the group exposed to 1000 mg/m^3^, the relative liver weights were increased in the male rats after 4 weeks, but not with statistical significance. Urine samples were examined from only 5 animals per sex and concentration. The coproporphyrin and uroporphyrin levels were increased in the urine of the animals in the high concentration group after 13 weeks, but without statistical significance. The authors suggested that the variations in the findings were caused by the instability of the porphyrins in the urine. In the high concentration group, 3 rats developed squamous cell metaplasia and focal hyperplasia in the respiratory epithelium that were characterized as reversible. The authors attributed these to irritation or the stress experienced by the animals during the experiments (Sasmore et al. 1983). It is difficult to derive a NOAEC because of the assumed instability of the porphyrins.

#### 5.2.2 Oral administration

The documentation published in 1990 included data for the effects induced by oral exposure. After exposure of rats to the 1,2,3-isomer, increased kidney weights, histological changes in the liver and thyroid gland (including degeneration of liver cells) as well as increased excretion of coproporphyrin with the urine at high doses (250 to 500 mg/kg body weight) were observed. After exposure of rats to the 1,2,4-isomer, increased organ weights (liver and kidneys), the induction of enzymes in the liver and, in the high dose groups (250 to 500 mg/kg body weight), necrosis and fatty changes in the liver and increased excretion of coproporphyrin with the urine were observed. In general, the trichlorobenzenes caused a decrease in body weights in animal models (Henschler 1992).

In a feeding study, groups of 10 male and 10 female Sprague Dawley rats were given 1,2,3-trichlorobenzene, 1,2,4-trichlorobenzene or 1,3,5-trichlorobenzene over a period of 13 weeks. The isomers were administered in concentrations of 0, 1, 10, 100 and 1000 mg/kg feed. This is equivalent to doses for the different isomers of 0, 0.08, 0.78 to 0.81, 7.6 to 7.8 and 78 to 82 mg/kg body weight and day for the male animals and 0, 0.11 to 0.13, 1.3 to 1.5, 12 to 17 and 101 to 146 mg/kg body weight and day for the female animals. At a concentration of 1000 mg/kg feed, increased liver weights and moderate histological changes in the liver and thyroid gland were observed in the male animals, irrespective of which of the 3 isomers was administered. After exposure to the 1,3,5-isomer, these changes were observed also in the kidneys. The authors derived a NOAEL (no observed adverse effect level) for all 3 isomers of 100 mg/kg feed (about 7 mg/kg body weight and day) (Côté et al. 1988). This study cannot be used to derive a limit value because its findings were not presented in sufficient detail. Most importantly, the histological findings were not evaluated quantitatively. Furthermore, the study did not investigate the most sensitive end point (disruption of porphyrin metabolism).

In the following, the data for toxic effects induced after oral administration are supplemented with the findings of other studies and summarized in Table 2.

In a 13-week feeding study, F344 rats (10 animals per sex and dose) were treated with 1,2,4-trichlorobenzene. The males were given 0, 14.6, 45.6 and 133.7 mg/kg body weight and day and the females 0, 17.0, 52.5 and 150.6 mg/kg body weight and day. The platelet count was increased with statistical significance in the male rats of the high dose group. The blood urea nitrogen levels were increased in both the males and the females of the high dose group. In addition, the total protein, albumin and calcium levels were increased in the male animals of this dose group. The aspartate aminotransferase activity was reduced in all treated males. In addition, the relative and absolute liver weights were increased with statistical significance in all treated males and in the females at doses of 52.5 mg/kg body weight and day and above. In the males of the high dose group, also the relative and absolute kidney weights were increased with statistical significance. At doses of 45.6 mg/kg body weight and day and above, the following effects were detected in the kidneys: dilated tubules, renal granular casts, hyaline droplets, interstitial nephritis and papillary mineral deposits. Kidney effects were not observed in the female animals. In the liver, centrilobular hepatocyte hypertrophy was found at doses of 45.6 mg/kg body weight and day and above (males) and at 150.6 mg/kg body weight and day (females) (CMA 1989).

In a 13-week study, groups of 10 male and 10 female B6C3F1 mice were given 1,2,4-trichlorobenzene with the feed. The males were treated with doses of 0, 67, 850 and 1222 mg/kg body weight and day and the females with 0, 86, 1183 and 1345 mg/kg body weight and day. At the end of the study, the body weights of the animals in the high dose group were decreased with statistical significance (by 9% in the males and by 8% in the females) in comparison with the values determined in the control group. In the low dose group, the body weight gains were reduced by 27% in the male animals. In the high dose group, the body weight gains were reduced by 40% in the males and by 33% in the females. Hepatocellular cytomegaly, karyomegaly, hepatocellular atrophy and cell degeneration were observed in the liver of the males at doses of 850 mg/kg body weight and day and above and in the females at doses of 1183 mg/kg body weight and day and above. In addition, the relative and absolute liver weights were increased in these animals. The total protein levels were increased in the male animals after exposure to 850 and 1222 mg/kg body weight and day and in the female animals after exposure to 1345 mg/kg body weight and day. Other findings in these dose groups were an increase in albumin and globulin levels as well as in alanine aminotransferase and sorbitol dehydrogenase activities (CMA 1989).

The feeding studies described in Section 5.7.2 were carried out with B6C3F1 mice and F344 rats to investigate possible carcinogenic effects induced by 1,2,4-trichlorobenzene. In rats, the liver weights were increased at 66.5 and 81.4 mg/kg body weight and day (males and females, respectively). The effects observed in the treated animals were hepatocyte hypertrophy, focal cystic degeneration and diffuse fatty liver changes. Papillary mineralization, hyperplasia of the transitional epithelium and nephropathy were observed in the kidneys of the male animals treated with 66.5 mg/kg body weight and day. In addition, all treated animals were found to have reduced body weights (CMA 1994 b). In mice, the liver weights were increased at 21 and 26 mg/kg body weight and day (males and females, respectively) and above. In addition, centrilobular hepatocytomegaly was observed at dose levels of 100 and 127 mg/kg body weight and day and above, respectively. Furthermore, the body weights of the treated animals were reduced. Liver adenomas and carcinomas were observed (CMA 1994 a). The neoplastic findings are shown in Section 5.7.

**Tab.2:** Effects of 1,2,4-trichlorobenzene after repeated oral exposure

Species, strain, number per group	Exposure	Findings	References
**rat**, F344, 10** ♀**, 10** ♂**	13 weeks, 1,2,4-trichlorobenzene in the feed: ♂: 0, 14.6, 45.6, 133.7 mg/kg body weight and day, ♀: 0, 17.0, 52.5, 150.6 mg/kg body weight and day, purity: > 98%	**14.6 mg/kg body weight and above (♂)**: aspartate aminotransferase activity ↓, relative and absolute liver weights ↑, **45.6 mg/kg body weight and above (♂)**: dilated tubules, renal granular casts, hyaline droplets, interstitial nephritis and papillary mineral deposition, centrilobular hepatocyte hypertrophy, **52.5 mg/kg body weight and above (♀)**: relative and absolute liver weights ↑, **133.7 mg/kg body weight (♂)**: platelet count ↑, blood urea nitrogen levels ↑, total protein, albumin, calcium levels ↑, relative and absolute kidney weights ↑, **150.6 mg/kg body weight (♀)**: centrilobular hepatocyte hypertrophy	CMA 1989
**rat**, F344, 50** ♀**, 50** ♂**	104 weeks, 1,2,4-trichlorobenzene in the feed: ♂: 0, 5.6, 19.4, 66.5 mg/kg body weight and day, ♀: 0, 6.9, 23.5, 81.4 mg/kg body weight and day, purity: 98.9%	**66.5 (♂) and 81.4 (♀) mg/kg body weight**: absolute and relative liver weights ↑, liver: diffuse, fatty changes, centrilobular hepatocytomegaly, focal cystic degeneration, kidneys: hyperplasia of the transitional epithelium, papillary mineralization	CMA 1994 b
**mouse**, B6C3F1, 10** ♀**, 10** ♂**	13 weeks, 1,2,4-trichlorobenzene in the feed: ♂: 0, 67, 850, 1222 mg/kg body weight and day, ♀: 0, 86, 1183, 1345 mg/kg body weight and day, purity: > 98%	**850 (♂) and 1183 (♀) mg/kg body weight and above**: liver: hepatocellular cytomegaly, karyomegaly, hepatocellular atrophy and cell degeneration, relative and absolute liver weights ↑, total protein** **↑, sorbitol dehydrogenase activity ↑, **1222 (♂) and 1345 (♀) mg/kg body weight and above**: body weights ↓, body weight gains ↓, albumin levels ↑, globulin levels ↑, alanine aminotransferase activity ↑	CMA 1989
**mouse**, B6C3F1, 50** ♀**, 50** ♂**	104 weeks, 1,2,4-trichlorobenzene in the feed: ♂: 0, 21, 100, 520 mg/kg body weight and day, ♀: 0, 26, 127, 573 mg/kg body weight and day, purity: 98.9%	**21 (♂) and 26 (♀) mg/kg body weight and above**: relative and absolute liver weights ↑, hepatomegaly, abdominal swelling, **100 (♂) and 127 (♀) mg/kg body weight and above**: liver: cell changes, centrilobular hepatocytomegaly, liver adenomas and liver carcinomas (see Section 5.7)	CMA 1994 a

#### 5.2.3 Dermal application

The documentation from 1990 (Henschler 1992) included data for the effects induced by absorption through the skin. A study investigating carcinogenic effects is discussed in detail in Section 5.7.2.

#### 5.2.4 Summary

In addition to the effects on the livers and kidneys of rats, the disruption of porphyrin metabolism is to be regarded as the most sensitive end point after exposure by inhalation and may be used for the derivation of a limit value for the workplace (see Section 6). Local effects on the respiratory tract or nose were not detected in any of the animal species exposed by inhalation. The most common effects induced in rats and mice after oral administration were damage to the liver and to the kidneys.

### 5.3 Local effects on skin and mucous membranes

The documentation published in 1990 and the addendum from 2002 (Greim 2002 b; Henschler 1992) included data for effects on the skin, mucous membranes and eyes. The relevant study findings are presented below again together with new findings.

#### 5.3.1 Skin

1,2,3-Trichlorobenzene (purity not specified) was mixed to a paste with water and applied semi-occlusively in amounts of 500 mg to the shaved skin of the flanks of 3 New Zealand White rabbits for 4 hours. Test readings were carried out 1, 24, 48 and 72 hours and 7 days after the end of treatment and the results were evaluated according to the Draize scale. The mean irritation index for redness (24, 48, 72 hours) was calculated to be 0.3 for 2 of the animals and 0 for the third animal (ECHA 2014; Henschler 1992).

Repeated application of undiluted 1,2,3-trichlorobenzene to the intact abdominal skin of rabbits caused redness and slight detachment of the skin. Moderate redness of the skin, mild oedema and traces of skin necrosis were found after application to the abraded skin (no other details; ATSDR 2014).

Application of 0.05 ml of a 75% or 95% solution of 1,2,4-trichlorobenzene to shaved skin on the shoulders of guinea pigs for 24 hours induced moderate to severe irritation in older animals and at most slight irritation in younger animals (no other details; ATSDR 2014; EC 2003).

After repeated occlusive application of 1 ml of undiluted 1,2,4-trichlorobenzene to the shaved skin of 4 male and 4 female rabbits (each application lasting for 6 hours on 3 consecutive days), spongiosis, acanthosis and parakeratosis were observed 7 days after the beginning of treatment. The same findings were observed after non-occlusive treatment of 5 female and 5 male guinea pigs and of 1 female and 1 male rabbit for 3 weeks. The animals were treated with 0.5 and 1 ml of undiluted 1,2,4-trichlorobenzene for 6 hours a day on 5 days a week (Brown et al. 1969).

In rabbits, repeated, non-occlusive application of undiluted substance (24 or 97 mg/kg body weight) or 0.2 ml of a 25% 1,2,4-trichlorobenzene solution in petroleum ether to the ventral skin of the ears (3 times a week, for 13 weeks) caused moderate to severe skin irritation such as mild to severe erythema, marked scaling and desquamation, encrustation with slight enlargement of the follicles, hair loss and scar formation. Rabbits that were treated with 0.2 ml of a 0.5% solution of 1,2,4-trichlorobenzene in petroleum ether (4.8 mg/kg body weight) developed slight redness of the skin in addition to mild scaling and desquamation of the skin. No effects were observed in control animals treated only with the solvent (Powers et al. 1975).

In a study, technical 1,2,4-trichlorobenzene (about 70% 1,2,4-trichlorobenzene and 30% 1,2,3-trichlorobenzene) was applied non-occlusively to the shaved dorsal skin of rabbits (about 100 cm^2^) at daily doses of 0, 30, 150 or 450 mg/kg body weight on 5 consecutive days per week for a total of 4 weeks. At the application site, all treated animals had cracked, scabbed and thickened skin in varying degrees of severity and size depending on the dose, fur that grew back dull and variable degrees of erythema, erosions and ulcers. The effects increased in severity with the dose. Histopathological changes were observed in the skin samples, including inflammation, focal erosion and ulcers as well as the accumulation of inflammatory cells with varying degrees of exudation. A number of rabbits had mild superficial oedema with slight fibrosis (Rao et al. 1982).

In a study of the carcinogenic effects induced by 1,2,4-trichlorobenzene in mice, application of the substance to the skin (0.03 ml of a 30% solution, 2 times a week, for 2 years; see Section 5.7.2) caused thickening of the skin, keratosis of the epidermis and inflammation (Henschler 1992; Yamamoto et al. 1982).

A single application of 1,2,4-trichlorobenzene led to only mild inflammation, whereas repeated application caused marked inflammation. Therefore, the isomer has been classified as irritating to the skin in the category Xi; R38 (EC 2003).

In a study with 1,3,5-trichlorobenzene, 500 mg (no other details) were applied to the shaved intact or abraded dorsal skin of groups of 6 rabbits. The application sites were covered (occlusive application). After 24 hours, erythema and oedema were observed in all treated animals. In 3 animals, marked redness was still noticeable 72 hours after application (Jorgenson et al. 1976). In another occlusive patch test, application to the intact or scarified flank skin of groups of 6 albino Himalayan rabbits caused slight irritation of the skin (irritation index 2.4 according to FDA guidelines) and changes to the skin (scaly, dry, etc.) (Henschler 1992).

#### 5.3.2 Eyes

1,2,3-Trichlorobenzene was instilled into the conjunctival sac of 3 rabbits in an amount of 100 mg. The eyes were rinsed 24 hours after application and examined 1 hour, 24, 48 and 72 hours and 7 days after treatment. In 2 of the 3 animals, mild clouding of the cornea (mean irritation indices at 24, 48 and 72 hours: 0.7, 0, 0.7), moderate to severe redness (1.3, 0.7, 1.3) and conjunctival swelling (0.3, 0, 0.7) with discharge (0, 0.3, 0.7) were observed. All effects had subsided 72 hours after administration (ECHA 2014; Henschler 1992).

In a study, a 10% solution of 1,2,3-trichlorobenzene in propylene glycol was instilled into the eyes of rabbits. The eyes were then rinsed with water. This induced acute pain and corneal injuries that healed within 24 hours while another effect, irritation of the conjunctiva, subsided only after 48 hours. Omitting the washing step likewise caused acute pain and irritation of the conjunctiva, but did not lead to injuries of the cornea. In these animals, however, the irritation subsided only after 48 hours (no other details; ATSDR 2014; EC 2003).

Pure 1,2,4-trichlorobenzene instilled into the rabbit eye caused conjunctivitis, chemosis and discharge in addition to severe, painful swelling of the eyelids accompanied by inflammation of the conjunctiva that lasted at least 48 hours. No adverse effects were observed in the cornea (Henschler 1992).

In a study, 100 mg of 1,3,5-trichlorobenzene was instilled into the conjunctival sac of 9 rabbits. The substance was washed out of the eyes of 2 groups of 3 animals; in one group after 30 seconds and in the other after 5 minutes. The examination was carried out 1 hour and 4, 24, 48, 72 and 96 hours after administration. Corneal damage was not found in the animals of the groups whose eyes were rinsed, while mild injuries were observed in the group whose eyes were not rinsed. The injuries had healed after 3 days. The effects on the iris that occurred in isolated cases had likewise subsided after 4 days. Conjunctival effects that varied in severity were observed in all treated animals. These included redness, oedema and discharge. The conjunctival effects observed in the group of animals whose eyes were rinsed after 30 seconds were no longer noticeable after 24 hours. In the group of animals whose eyes were rinsed after 5 minutes, however, the last effects disappeared only after 3 days. The effects in the group whose eyes were not rinsed subsided only after 7 days (Jorgenson et al. 1976).

#### 5.3.3 Summary

The trichlorobenzenes cause mild to moderate irritation of the skin and eyes of rabbits and guinea pigs; however, these effects are reversible. Manifest injuries occurred only at higher concentrations or after longer periods of exposure and repeated application. These are probably due to the degreasing effects of the substances.

### 5.4 Allergenic effects

#### 5.4.1 Sensitizing effects on the skin

The trichlorobenzenes were last evaluated for their skin sensitizing potential in 1990. The evaluation of 1,2,4-trichlorobenzene and 1,3,5-trichlorobenzene was based on negative findings obtained in guinea pigs; however, only 0.1% formulations had been studied. No new animal studies are available for 1,2,4-trichlorobenzene and 1,3,5-trichlorobenzene.

1,2,3-Trichlorobenzene has in the meantime been investigated in a modified local lymph node assay (LLNA-IMDS, “integrated method for the discrimination of skin reactions” according to Vohr) (ECHA 2014). This method evaluates the sensitizing potential of a substance based on the proliferation of lymph node cells and an increase in lymph node weights. In addition, the irritant potential of a substance is evaluated by determining ear thickness and mass. The interpretation of all parameters makes it possible to differentiate between the sensitizing and irritating potential of a test substance (Vohr et al. 2000).

The LLNA-IMDS was carried out with 24 female NMRI mice (Hsd Win:NMRI). Each treatment group was composed of 6 animals (2%, 10% and 50% 1,2,3-trichlorobenzene in acetone/olive oil (4:1)) with another 6 animals forming a control group. The stimulation indices (SI) for cell proliferation were 0.94, 1.20 and 1.73. The established threshold values for positivity (index ≥ 1.4 in comparison with the values for the controls or > 1.5 (Basketter et al. 2012)) were exceeded in the high dose group (50%). The EC1.4 value (the concentration at which cell proliferation is increased 1.4-fold) was estimated to be 25.09% 1,2,3-trichlorobenzene. The indices for ear swelling and mass did not deviate significantly from the control values. Thus, the positive results obtained for cell proliferation in the LLNA were apparently not affected by the irritant effects of 1,2,3-trichlorobenzene.

#### 5.4.2 Sensitizing effects on the airways

There are no data available.

### 5.5 Reproductive and developmental toxicity

The data for fertility and for toxic effects on development induced by the trichlorobenzenes were described in the documentation from 1990 and in the addendum from 2007 (Greim 2007; Henschler 1992). The results are summarized below together with a description of recent data.

#### 5.5.1 Fertility

In a multi-generation study, 100 pregnant rats (strain not specified, data only for the breeder) were given commercial feed ad libitum and untreated drinking water up to the birth of the F0 generation and were then treated with 1,2,4-trichlorobenzene concentrations of 0, 25, 100 or 400 mg/l drinking water. In the F0 generation, this was equivalent to doses of 2.5, 8.9 and 33 mg/kg body weight and day for the male animals and 3.7, 14.8 and 53.6 mg/kg body weight and day for the female animals. A fifth group was given 0.125% Tween 20 with the drinking water. This served as a solubilizer because 1,2,4-trichlorobenzene is insoluble in water at these concentrations. The animals were treated continuously until the F2 generation were completely weaned. Each generation was composed of 17 to 23 offspring. The test parameters were fertility, offspring size and sex, body weights and viability. The animals of the F0 and F2 generations were examined at death as well as individual animals at the age of 27 and 95 days. The animals of the F1 generation were examined at death or at an age of 95 days. At each of the mentioned time points, blood samples from 10 animals per group were analysed (glucose, urea, blood urea nitrogen, creatinine, electrolytes, uric acid, calcium, phosphate, cholesterol, triglycerides, bilirubin, the activities of alkaline phosphatase, alanine aminotransferase, lactate dehydrogenase and creatine phosphokinase, total protein, globulin and albumin concentrations). The following organ weights were determined: liver, kidneys, adrenal glands, uterus, lungs, heart and ovaries. In addition, a histological examination of the liver and kidneys of the control group and the high dose group of the F1 generation was carried out. No adverse effects on the fertility of the animals were detected. Other than an increase in the weights and sizes of the adrenal glands observed in high-dose males and females of the F0 and F1 generations at the age of 95 days, none of the generations were found to have any effects in the blood or the examined organs. The authors interpreted these findings in the adrenal glands as substance-induced effects (ATSDR 2014; Henschler 1992; Robinson et al. 1981; US EPA 2020 b). On this basis, the NOAEL for effects on fertility was 33 mg/kg body weight and day in the males and 53.6 mg/kg body weight and day in the females. The NOAEL for parental toxicity (treated F1 generation) was 8.9 mg/kg body weight and day in the males and 14.8 mg/kg body weight and day in the females.

In inhalation studies, no effects on the reproductive organs of Sprague Dawley rats, New Zealand White rabbits or beagle dogs were observed after exposure to 1,2,4-trichlorobenzene up to a concentration of 100 ml/m^3^ (760 mg 1,2,4-trichlorobenzene/m^3^) (Henschler 1992; Kociba et al. 1981). Negative findings were obtained in the reproductive organs of CD rats after exposure to 1,3,5-trichlorobenzene up to a maximum concentration of 1000 mg/m^3^ (Henschler 1992; Sasmore et al. 1983). There are no data available from inhalation studies for the effects of 1,2,3-trichlorobenzene on fertility. The negative findings obtained in the reproductive organs were confirmed for all 3 trichlorobenzene isomers by studies with oral exposure of Sprague Dawley rats. The animals were exposed to 1,2,4-trichlorobenzene (82 mg/kg body weight for males, 101 mg/kg body weight for females), 1,2,3-trichlorobenzene (78 mg/kg body weight for males, 113 mg/kg body weight for females) or 1,3,5-trichlorobenzene (82 mg/kg body weight for males, 146 mg/kg body weight for females) (Côté et al. 1988; Henschler 1992). In a feeding study with the administration of a maximum dose of 1,2,4-trichlorobenzene of 150.6 mg/kg body weight and day to F344 rats for a period of 14 days, no effects on the reproductive organs were found (ATSDR 2014). Likewise, in studies with male and female B6C3F1 mice, no effects were observed in the reproductive organs up to 1,2,4-trichlorobenzene doses of 1222 and 1345 mg/kg body weight and day, respectively, and a period of exposure of 13 weeks (ATSDR 2014). No effects on these target organs were observed in two 2-year feeding studies with F344 rats and B6C3F1 mice (CMA 1994 a, b). These studies are described in Section 5.2 and Section 5.7.

#### 5.5.2 Developmental toxicity

Groups of pregnant Sprague Dawley rats (at least 6 animals per group) were given 1,2,4-trichlorobenzene dissolved in corn oil in doses of 0, 36, 120, 360 or 1200 mg/kg body weight and day from gestation days 9 to 13 and sacrificed on day** **14. The livers of the dams were weighed and examined histologically. The enzyme levels in liver homogenates were analysed. The animals of the control group and the dose group treated with 360 mg/kg body weight and day were examined for implantations and resorptions in the uterus. Living foetuses were examined superficially (size, heartbeat, somitogenesis). Two of the 9 animals in the 360 mg/kg group and 6 of the 6 animals in the 1200 mg/kg group died during the experiment. The body weight gains of the animals of the 360 mg/kg group were decreased with statistical significance. No effects on the maternal liver weights, the microsomal protein content in the liver and the relative liver weights were observed. In the 360 mg/kg group, NADPH cytochrome c reductase activity was increased in the dams. In the groups exposed to 120 and 360 mg/kg body weight and day, hepatic CYP levels were significantly increased and a series of other liver enzymes were induced. Hepatocellular hypertrophy was found in 1 of the 9 rats in the 120 mg/kg group and moderate hypertrophy in 7 of the 8 rats in the 360 mg/kg group. A higher incidence of resorptions, increased lethality and teratogenic effects were not observed. Marked delays in foetal development were found only in the 360 mg/kg group. These included reduced head and crown–rump lengths, a decreased number of somites and decreased protein contents (Henschler 1992; Kitchin and Ebron 1983). The current OECD Test Guideline 414 recommends the use of a sufficient number of dams to ensure that there are about 20 animals with implantation sites at necropsy. With only 6 animals per group, a comparatively small number of animals was used. Treatment did not quite cover the entire phase of organogenesis in rats (gestation days 5 to 15). As described above, the protocol included only a superficial examination of the foetuses by stereo microscope. The external as well as the visceral and skeletal changes were not examined. The study is not suitable for evaluating the toxic effects of 1,2,4-trichlorobenzene on development due to its considerable shortcomings.

Groups of 13 to 14 Sprague Dawley rats were given gavage doses of 1,2,3-trichlorobenzene or 1,3,5-trichlorobenzene of 0, 150, 300 or 600 mg/kg body weight and day from gestation days 6 to 15. Using the same procedure, other groups of animals were given 1,2,4-trichlorobenzene doses of 0, 75, 150 or 300 mg/kg body weight and day. The additional dose of 75 mg/kg body weight was included because preliminary studies had shown that the 1,2,4-isomer was more toxic for the dams. The animals were sacrificed and examined on gestation day 22. After exposure to 1,2,4-trichlorobenzene, 1,2,3-trichlorobenzene and 1,3,5-trichlorobenzene, the absolute and relative liver weights were increased with statistical significance in the high dose group, but no effects were observed in other organs. After exposure to 1,2,4-trichlorobenzene, oxidase activity was increased in the dams of the 150 and 300 mg/kg groups. The haemoglobin levels and haematocrit values in the blood of the dams were reduced after exposure to 1,2,4-trichlorobenzene, 1,2,3-trichlorobenzene and 1,3,5-trichlorobenzene at 300 mg/kg body weight and day. After exposure to 1,2,4-trichlorobenzene, the follicular cells in the thyroid glands of the foetuses of the high dose group were reduced in size and slight hepatic lesions (periportal cytoplasmic eosinophilia and mild anisokaryosis of the nuclei) were observed at 300 mg/kg body weight and day. No effects were observed on the ovaries or uterus of the animals. Overall, no dose-dependent embryotoxic or teratogenic effects were found. The incidence of resorptions was significantly increased only at a 1,3,5-trichlorobenzene dose of 300 mg/kg body weight; this finding was based on an animal with 12 resorption sites. As the incidence of resorptions was not increased in any other dose group, the authors concluded that this was not a substance-induced effect (Black et al. 1988; Greim 2007; Henschler 1992; US EPA 2009 b). Effects on the eyes of the foetuses are regarded as questionable findings because the samples for analysis were insufficiently preserved and the results were not reported in sufficient detail (EC 2003). On the basis of these study findings, the NOAEL for developmental toxicity was 300 mg/kg body weight and day after exposure to 1,2,4-trichlorobenzene and 600 mg/kg body weight and day (the highest dose in each case) after exposure to 1,2,3-trichlorobenzene and 1,3,5-trichlorobenzene. Teratogenicity was not induced by any of the 3 isomers. The study, which was published in 1988, does not comply with the test guidelines used today, but does fulfil the requirements for the scope of investigation (external examination of all foetuses, examination of the skeletons of 2/3 of the foetuses and visceral examination of 1/3 of the foetuses). The number of tested animals is not as high as recommended by current OECD test guidelines. However, all 3 isomers were examined, which increases the number of foetuses studied and, as the isomers are metabolized along similar pathways, the study is considered relevant for the evaluation of developmental toxicity.

In the multi-generation study in rats described above, effects on newborn body weights, litter size and survival were not found in any of the generations up to the high dose of 1,2,4-trichlorobenzene of 53.6 mg/kg body weight and day (ATSDR 2014; Henschler 1992; Robinson et al. 1981; US EPA 2009 b; see Section 5.5.1). For this reason, the NOAEL for perinatal toxicity is 53.6 mg/kg body weight and day, the highest dose tested.

Several publications reported the development of a screening method for teratogenicity in which 25 pregnant CD-1 mice were given gavage doses of 1,2,4-trichlorobenzene of 0 or 130 mg/kg body weight and day from gestation days 8 to 12. The parameters investigated were survival of the dams, body weights, pregnancy incidence, uterus and the number of living and dead foetuses. No substance-induced effects were observed in the treated animals (US EPA 2020 b). Screening studies are not suitable for the evaluation of developmental toxicity because they do not include a complete evaluation of teratogenicity.

### 5.6 Genotoxicity

Studies of genotoxicity induced by the trichlorobenzene isomers were discussed in the documentation from 1990 (Henschler 1992) and in the addendum on 1,2,4-trichlorobenzene (Greim 2000). The data for genotoxicity are reviewed again below and presented together with recently published study findings.

#### 5.6.1 In vitro

##### 5.6.1.1 Bacteria and yeasts

Studies of the genotoxic effects induced by the 3 isomers in vitro are shown in Table 3.

In an umu test system used to detect the induction of an SOS response in Salmonella typhimurium TA1535 containing plasmid pSK1002, no evidence of the induction of DNA damage by the isomers 1,2,3-trichlorobenzene and 1,3,5-trichlorobenzene was found (Ono et al. 1992). Indicator tests (tests for differential killing) using Escherichia coli W3110/p3478 or Bacillus subtilis H17/M45 likewise did not provide evidence of DNA-damaging effects induced by 1,3,5-trichlorobenzene (Jorgenson et al. 1976). DNA-damaging effects were observed in the umu test with Salmonella typhimurium TA1535 after incubation with 1,2,4-trichlorobenzene in a concentration of 0.1 mg/ml. These effects increased in severity with the length of the incubation period. However, the same results were not observed in another umu test after incubation for 4 hours (Ono et al. 1991, 1992). A test for differential killing using Bacillus subtilis H17/M45 likewise found that 1,2,4-trichlorobenzene may induce genotoxic effects (Matsui et al. 1989). Overall, these results are regarded as inconsistent because the findings that can be interpreted as evidence of genotoxicity were observed once only without metabolic activation (umu test) or only with metabolic activation (test for differential killing). Furthermore, the test used for the study of Matsui et al. (1989) was not documented in sufficient detail; for example, the S9 mix was neither identified nor described.

1,3,5-Trichlorobenzene did not induce mutagenic effects in mutagenicity tests with Escherichia coli WP2 (Jorgenson et al. 1976). The 3 isomers were not mutagenic in Salmonella mutagenicity tests. The tests were carried out with the strains TA92, TA94, TA98, TA100, TA1535 and TA1537 (Haworth et al. 1983; Henschler 1992; Miyata et al. 1981; Schoeny et al. 1979). Studies that were not yet included in the earlier documentation or in the addenda likewise reported negative results in Salmonella mutagenicity tests carried out with 1,2,3-trichlorobenzene and 1,2,4-trichlorobenzene in the strains TA97 and TA100 (Kubo et al. 2002) and with 1,2,4-trichlorobenzene additionally in the strains TA1535, TA1537 and TA1538 (Ethyl Corporation 1975).

1,2,4-Trichlorobenzene did not induce mitotic recombination in D3 yeast cells (Ethyl Corporation 1975). 1,3,5-Trichlorobenzene increased the incidence of mitotic recombination in these cells; however, this was not dependent on the concentration (Jorgenson et al. 1976).

##### 5.6.1.2 Mammalian cells

1,2,4-Trichlorobenzene did not induce genotoxicity in indicator tests used to detect DNA repair in rat hepatocytes (Henschler 1992; Shimada et al. 1983; Williams et al. 1989).

No chromosomal aberrations were found in the kidney and ovarian cells of Chinese hamsters after exposure to 1,2,3-trichlorobenzene (Henschler 1992; Ishidate et al. 1988; McElroy et al. 2003). Chromosomal aberrations were likewise not induced in the lung and ovarian cells of Chinese hamsters after exposure to 1,2,4-trichlorobenzene or 1,3,5-trichlorobenzene (Henschler 1992; Ishidate et al. 1988).

Radioactively labelled 1,2,4-trichlorobenzene (0.1 mM) was incubated with microsomal rat liver protein (0.5 and 1 mg protein). The substance was metabolized to form trichlorophenols and trichlorohydroquinones. After incubation, 9.4% and 17% of the applied radioactivity, respectively, had formed covalent bonds with the proteins. Following the addition of 1 mg of calf thymus DNA, 0.5% of the applied radioactivity formed covalent bonds with the DNA. Lipid peroxidation did not occur. The authors interpreted this as evidence that chlorinated benzenes probably do not undergo extensive redox cycling after the quinones are formed (den Besten et al. 1991 a). However, this study did not include information relating to the degree to which the DNA was purified prior to testing. For this reason, it cannot be ruled out that the bound radioactivity may be attributable to protein impurities in the DNA sample. It is therefore possible that the radio­actively labelled degradation products (such as the quinones) may have previously formed bonds with the proteins.

**Tab.3:** Genotoxicity of 1,2,3-trichlorobenzene, 1,2,4-trichlorobenzene and 1,3,5-trichlorobenzene in vitro

End point	Test system	Concentration [µg/plate]^a)^	Effective concen­tration [µg/plate]^a)^	Cytotoxicity [µg/plate]^a)^	**Results**		**References**
					**–m. A.**	**+m. A.**	
**1,2,3-trichlorobenzene**							
indicator test bacteria, DNA damage	S. typhimurium TA1535 containing plasmid pSK1002, umu test for SOS repair	0.1 mg/ml, 4 hours		not specified	–	–	Ono et al. 1992
**1,2,4-trichlorobenzene**							
indicator test bacteria, DNA damage	S. typhimurium TA1535 containing plasmid pSK1002, umu test for SOS repair	0.1 mg/ml, 4 hours or 2–20 hours	0.1 mg/ml	not specified	– after 4 hours + after 2 hours ++ after 4–20 hours	–	Ono et al. 1991, 1992
indicator test bacteria, differential killing	B. subtilis H17 (arg^–^, trp^–^, recE^+^) B. subtilis M45 (arg^–^, trp^–^, recE^–^)	not specified	138 µg/ml		–	+	Matsui et al. 1989
**1,3,5-trichlorobenzene**							
indicator test bacteria, DNA damage	S. typhimurium TA1535 containing plasmid pSK1002, umu test for SOS repair	0.1 mg/ml, 4 hours		not specified	–	–	Ono et al. 1992
indicator test bacteria, differential killing	E. coli W3110 E. coli p3478 (polA–)	100, 500, 1000		–	–	n. t.	Jorgenson et al. 1976
	B. subtilis H17 B. subtilis M45 (rec–)	100, 500, 1000		–	–	n. t.	
**1,3,5-trichlorobenzene**							
gene mutation Escherichia coli	E. coli WP2	5–5000		not specified	–	–	Jorgenson et al. 1976
**1,2,3-trichlorobenzene**							
gene mutation Salmonella typhimurium	S. typhimurium TA98	3.3–333.3		≥ 100 (–m. A.)	–	–	Haworth et al. 1983
	S. typhimurium TA100	3.3–333.3		≥ 100 (–m. A.)	–	–	
	S. typhimurium TA1535	3.3–333.3		≥ 100 (–m. A.)	–	–	
	S. typhimurium TA1537	3.3–333.3		≥ 100 (–m. A.)	–	–	
	S. typhimurium TA92	30–3000		≥ 100 (–m. A.), 3000 (+m. A.)	–	–	Miyata et al. 1981
	S. typhimurium TA94	30–3000		≥ 100 (–m. A.), 3000 (+m. A.)	–	–	
	S. typhimurium TA98	30–3000		≥ 100 (–m. A.), 3000 (+m. A.)	–	–	
	S. typhimurium TA100	30–3000		≥ 100 (–m. A.), ≥ 1000 (+m. A.)	–	–	
	S. typhimurium TA1535	30–3000		≥ 100 (–m. A.), 3000 (+m. A.)	–	–	
	S. typhimurium TA1537	30–3000		≥ 100 (–m. A.), ≥ 1000 (+m. A.)	–	–	
	S. typhimurium TA98	0.01; 1 mM		not specified	–	–	Kubo et al. 2002
	S. typhimurium TA100	0.01; 1 mM		not specified	–	–	
**1,2,4-trichlorobenzene**							
gene mutation Salmonella typhimurium	S. typhimurium TA98	3.3–333.3		≥ 100 (–m. A.)	–	–	Haworth et al. 1983
	S. typhimurium TA100	3.3–333.3		≥ 100 (–m. A.)	–	–	
	S. typhimurium TA1535	3.3–333.3		≥ 100 (–m. A.)	–	–	
	S. typhimurium TA1537	3.3–333.3		≥ 100 (–m. A.)	–	–	
	S. typhimurium TA92	30–3000		≥ 100 (–m. A.), 3000 (+m. A.)	–	–	Miyata et al. 1981
	S. typhimurium TA94	30–3000		≥ 100 (–m. A.), 3000 (+m. A.)	–	–	
	S. typhimurium TA98	30–3000		≥ 100 (–m. A.), 3000 (+m. A.)	–	–	
	S. typhimurium TA100	30–3000		≥ 100 (–m. A.), 3000 (+m. A.)	–	–	
	S. typhimurium TA1535	30–3000		≥ 100 (–m. A.), ≥ 1000 (+m. A.)	–	–	
	S. typhimurium TA1537	30–3000		≥ 100 (–m. A.), 3000 (+m. A.)	–	–	
	S. typhimurium TA98	102–140 000		≥ 1599	–	–	Schoeny et al. 1979
	S. typhimurium TA100	102–140 000		≥ 1599	–	–	
	S. typhimurium TA1535	102–140 000		≥ 1599	–	–	
	S. typhimurium TA1537	102–140 000		≥ 1599	–	–	
	S. typhimurium TA98	administered in a 5-fold concentra­tion as specified by test guidelines (no other data)		not specified	–	–	Henschler 1992
	S. typhimurium TA100	see above		not specified	–	–	
	S. typhimurium TA1535	see above		not specified	–	–	
	S. typhimurium TA1537	see above		not specified	–	–	
	S. typhimurium TA1538	see above		not specified	–	–	
	S. typhimurium TA98	0.01, 1 mM		not specified	–	–	Kubo et al. 2002
	S. typhimurium TA100	0.01, 1 mM		not specified	–	–	
	S. typhimurium TA1535	1, 10, 100, 500, 750, 1000, 5000		≥ 500 (–m. A.)	–	–	Ethyl Corporation 1975
	S. typhimurium TA1537	1, 10, 100, 500, 750, 1000, 5000		≥ 1000	–	–	
	S. typhimurium TA1538	1, 10, 100, 500, 750, 1000, 5000		≥ 500	–	–	
**1,3,5-trichlorobenzene**							
gene mutation Salmonella typhimurium	S. typhimurium TA198	33.3–3333.3		not specified	–	–	Haworth et al. 1983
	S. typhimurium TA100	33.3–3333.3		not specified	–	–	
	S. typhimurium TA1535	33.3–3333.3		not specified	–	–	
	S. typhimurium TA1537	33.3–3333.3		not specified	–	–	
	S. typhimurium TA92	30–3000		–	–	–	Miyata et al. 1981
	S. typhimurium TA94	30–3000		–	–	–	
	S. typhimurium TA98	30–3000		–	–	–	
	S. typhimurium TA100	30–3000		–	–	–	
	S. typhimurium TA1535	30–3000		–	–	–	
	S. typhimurium TA1537	30–3000		–	–	–	
	S. typhimurium TA98	100–10 000		10 000	–	–	Henschler 1992
	S. typhimurium TA100	100–10 000		10 000	–	–	
	S. typhimurium TA2637	100–10 000		10 000	–	–	
	S. typhimurium TA98	5–5000		not specified	–	–	Jorgenson et al. 1976
	S. typhimurium TA100	5–5000		not specified	–	–	
	S. typhimurium TA1535	5–5000		not specified	–	–	
	S. typhimurium TA1537	5–5000		not specified	–	–	
	S. typhimurium TA1538	5–5000		not specified	–	–	
**1,3,5-trichlorobenzene**							
gene mutation yeast	mitotic recombination S. cerevisiae D3	0.05%–5%	not specified	not specified	(+) > 3-fold incidence, not de­pend­­ent on con­centra­tion	(+) > 3-fold incidence, not de­pend­ent on con­centra­tion	Jorgenson et al. 1976
**1,2,4-trichlorobenzene**							
gene mutation yeast	mitotic recombination S. cerevisiae D3	0.02%, 0.002% (no other data)		0.02%	–	–	Ethyl Corporation 1975
**1,2,4-trichlorobenzene**							
indicator test mammalian cells	DNA repair rat hepatocytes	0.05–1 mM		not specified	–	n. t.	Henschler 1992
	DNA repair F344 rat hepatocytes	0.081 mM		–	–	n. t.	Williams et al. 1989
	DNA repair F344 rat hepatocytes, ♂	10^–5^%–1%		≥ 0.01%	–	n. t.	Shimada et al. 1983
**1,2,3-trichlorobenzene**							
chromosomal aberrations	Chinese hamster cells BHK21 (kidneys)	0.0157–0.125 mg/ml, 24 hours		not specified	–	–	Henschler 1992
	Chinese hamster cells CHL (lungs)	62.5 µg/ml, 48 hours		not specified	–	n. t.	Ishidate et al. 1988
	Chinese hamster cells CHL (lungs)	secondary evalua­tion of published data; not specified: negative and positive controls, con­centration range,3 hours ± m. A. and substance, then 21 hours	analysis of chromosomal aberrations: “gaps, breaks, exchanges”; findings evaluated as negative if < 5% aberrant cells	cell proliferation not reduced by more than 50% in the tested range	–	–	McElroy et al. 2003
	Chinese hamster cells CHO (ovaries)	see above	see above	see above	–	–	
**1,2,4-trichlorobenzene**							
chromosomal aberrations	Chinese hamster cells CHO (ovaries)	0.0313–0.125 mg/ml, 24 hours		not specified	–	n. t.	Henschler 1992
chromosomal aberrations	Chinese hamster cells CHL (lungs)	62.5 µg/ml, 48 hours		not specified	–	n. t.	Ishidate et al. 1988
**1,3,5-trichlorobenzene**							
chromosomal aberrations	Chinese hamster cells CHO (ovaries)	0.0157–0.0625 mg/ml, 24 hours		not specified	–	n. t.	Henschler 1992
chromosomal aberrations	Chinese hamster cells CHL (lungs)	62.5 µg/ml, 48 hours		not specified	–	n. t.	Ishidate et al. 1988

–: negative results; +: positive results; –m. A.: without metabolic activation; +m. A.: with metabolic activation; n. t.: not tested

a) unless specified otherwise, concentrations in [µg/plate]

#### 5.6.2 In vivo

Studies of the genotoxic effects induced by the 3 isomers in vivo are shown in Table 4.

##### 5.6.2.1 Drosophila

1,3,5-Trichlorobenzene yielded negative results in the SLRL test (X-chromosome-linked recessive lethal mutations), a germ cell test with Drosophila melanogaster (Zimmering et al. 1985).

##### 5.6.2.2 Tests for clastogenicity

In micronucleus tests with NMRI mice, positive findings were obtained for all 3 isomers in the bone marrow after administration by intraperitoneal injection. Groups of 5 male mice were given one of the isomers in 2 intraperito­neal doses of 0, 105, 210, 315 or 420 mg/kg body weight. The interval between the doses was 24 hours. At all doses, the number of micronuclei in the polychromatic cells was increased 6 hours after administration of the last dose (30 hours after the first injection) in comparison with the numbers determined in the controls. 1000 polychromatic erythrocytes were counted per animal (Mohtashamipur et al. 1987). However, the study had considerable methodological shortcomings: only a time-lagged control group for 6 test substances was used instead of a concurrent control group, the range of historical control data was not specified and the results of cytotoxicity assays were not given. In other micronucleus tests in groups of 3 Swiss CD1 mice, the numbers of micronuclei in the bone marrow were increased after intraperitoneal injection of 1,2,3-trichlorobenzene or 1,2,4-trichlorobenzene at a dose of 500 mg/kg body weight or 1,3,5-trichlorobenzene at 650 mg/kg body weight (2 doses administered at an interval of 24 hours). The ratio of polychromatic to normochromatic erythrocytes remained unchanged in the treated animals in comparison with that in the control animals (Parrini et al. 1990). Overall, the results of both micronucleus tests are regarded as of only limited validity. The number of test animals used for the study of Parrini et al. (1990) was too small and the study of Mohtashamipur et al. (1987) was restricted by the methodological shortcomings described above. Neither of the studies used the time interval of 24 hours recommended by OECD Test Guideline 487. Another micronucleus test was carried out to verify the findings of Mohtashamipur et al. (1987). In this study, the animals were treated with a single oral dose of 0, 100, 330 or 1000 mg/kg body weight. The bone marrow cells of 18 female and 18 male NMRI mice per dose group were analysed. Negative results were obtained after 24 and 48 hours. At 72 hours, the number of micronuclei was increased with statistical significance in comparison with the number determined in the controls; however, according to the authors, the control value was unusually low (0.01%). When compared with the historical control data (0.07%), the results were evaluated as negative (CCR Cytotest Cell Research 1990).

**Tab.4:** Genotoxicity of 1,2,3-trichlorobenzene, 1,2,4-trichlorobenzene and 1,3,5-trichlorobenzene in vivo

End point	Test system	Dose	Results	Comments	References
**1,3,5-trichlorobenzene**					
SLRL X-chromosome-linked recessive lethal mutations	Drosophila melanogaster	5000 and 40 000 mg/l in the feed	–		Zimmering et al. 1985
**1,2,3-trichlorobenzene**					
micronucleus test	mouse (NMRI), 5 ♂ per dose, bone marrow	0, 125, 250, 375, 500 mg/kg body weight, 2× intraperitoneal in corn oil at an interval of 24 hours, examination 6 hours after last dose	(+)	weakly positive (erythrocytes from the femoral bone marrow), control group for only 1 treatment time point (not all animals treated on the same day), no data for cytotoxicity, evaluation of 1000 PCE	Mohtashamipur et al. 1987
	mouse (Swiss-CD1), 3 ♂ per dose, bone marrow	0, 500 mg/kg body weight, 2× intraperitoneal in olive oil at an interval of 24 hours, examination 6 hours after last dose; positive control with benzene (2× 528 mg/kg body weight, administration the same as for 1,2,3-trichloro­benzene)	+	control: 1.16/3000 PCE; samples with solvent: 1.88/3000 PCE; samples with 1,2,3-trichlorobenzene: 3.22/3000 PCE; positive control benzene: 5.13/3000 PCE ratio of PCE to NCE unchanged in animals treated with 1,2,3-trichlorobenzene, results based on the evaluation of 1000 PCE	Parrini et al. 1990
**1,2,4-trichlorobenzene**					
micronucleus test	mouse (NMRI), 5 ♂ per dose, bone marrow	0, 105, 210, 315, 420 mg/kg body weight, 2× intraperitoneal in corn oil at an interval of 24 hours, examination 6 hours after last dose	(+)	weakly positive (erythrocytes from the femoral bone marrow), control group for only 1 treatment time point (not all animals treated on the same day), no data for cytotoxicity, evaluation of 1000 PCE	Mohtashamipur et al. 1987
	mouse (Swiss-CD1), 3 ♂ per dose, bone marrow	0, 500 mg/kg body weight, 2× intraperitoneal in olive oil at an interval of 24 hours, examination 6 hours after last dose; positive control with benzene (2× 528 mg/kg body weight, administration the same as for 1,2,4-trichlorobenzene)	+	control: 1.16/3000 PCE; samples with solvent: 1.88/3000 PCE; samples with 1,2,4-trichlorobenzene: 3.66/3000 PCE; positive control benzene: 5.13/3000 PCE ratio of PCE to NCE unchanged in animals treated with 1,2,4-trichlorobenzene, results based on the evaluation of 1000 PCE	Parrini et al. 1990
	mouse (NMRI), 18 animals per sex and dose, bone marrow	0, 100, 330, 1000 mg/kg body weight, oral in polyethylene glycol, examination after 24, 48, 72 hours	–	ratio of PCE to NCE only slightly decreased; negative results at 24 and 48 hours, incidence increased with statistical significance in the groups given 1000 mg/kg body weight in comparison with that of the concurrent control (0.07% compared with 0.01%) only after 72 hours; however, according to the authors, 0.01% is unusually low for controls. Historical controls had values of 0.073% (range: 0.04%–0.12%), therefore evaluated as negative	CCR Cytotest Cell Research 1990
**1,3,5-trichlorobenzene**					
micronucleus test	mouse (NMRI), 5 ♂ per dose, bone marrow	0, 215.5, 425, 637.5, 850 mg/kg body weight, 2× intraperitoneal in corn oil at an interval of 24 hours, examination 6 hours after last dose	(+)	weakly positive (erythrocytes from the femoral bone marrow), control group for only 1 treatment time point (not all ani­mals treated on the same day), no data for cytotoxicity, evaluation of 1000 PCE	Mohtashamipur et al. 1987
	mouse (Swiss-CD1), 3 ♂ per dose, bone marrow	0, 650 mg/kg body weight, 2× intraperitoneal in olive oil at an interval of 24 hours, examination 6 hours after last dose; positive control with benzene (2× 528 mg/kg body weight, administration the same as for 1,3,5-trichlorobenzene)	+	control: 1.16/3000 PCE; samples with solvent: 1.88/3000 PCE; samples with 1,3,5-trichlorobenzene: 2.42/3000 PCE; positive control benzene: 5.13/3000 PCE, ratio of PCE to NCE unchanged in animals treated with 1,3,5-trichlorobenzene, results based on the evaluation of 1000 PCE	Parrini et al. 1990

–: negative results; +: positive results; (+): weak positive results; NCE: normochromatic erythrocytes; PCE: polychromatic erythrocytes

#### 5.6.3 Summary

Overall, the data do not demonstrate that the trichlorobenzenes are mutagenic in bacteria in vitro. Negative results were obtained for genotoxic and clastogenic effects in mammalian cells in vitro. The only positive findings were the formation of bonds with microsomal proteins following incubation with the 1,2,4-isomer and weak DNA binding in cell-free systems with the addition of calf thymus DNA. However, the evidence does not show whether bonds were actually formed with the DNA or whether this finding is to be attributed to protein binding. In an indicator test (umu test) in vitro, positive findings were obtained without metabolic activation only under cytotoxic conditions. A test for differential killing in Bacillus subtilis yielded positive results with metabolic activation. All other genotoxicity studies in vitro, particularly those in mammalian cells, yielded negative results. In vivo studies in Drosophila reported negative findings for genotoxic effects. Positive findings were obtained for all 3 isomers in micronucleus tests in NMRI and Swiss CD1 mice; however, these tests are regarded to be invalid. Both studies have a number of methodological shortcomings and, by today’s standards, are not unreservedly suitable for use in an evaluation. Therefore, it is important to keep in mind that negative results were obtained in a valid micronucleus test with oral administration to NMRI mice. Thus, based on valid study findings, the trichlorobenzenes are not expected to induce clastogenic effects in vivo. This conclusion is supported also by the fact that positive findings were not obtained for the induction of clastogenic effects in vitro. Overall, the trichlorobenzenes are not considered genotoxic.

### 5.7 Carcinogenicity

The data for the carcinogenic effects induced by the trichlorobenzenes were presented in the documentation published in 1990 and in the addendum from 1996 (Greim 2000; Henschler 1992). Below, the data are re-evaluated, summarized and presented together with recent study findings.

#### 5.7.1 Short-term studies

In an in vitro study carried out to investigate cell transformation, rat liver epithelial cells (ARL) were exposed 3 times to 1,2,4-trichlorobenzene concentrations of 1.46, 14.6, 146.34 or 1463.4 µg/ml for 0, 2 or 4 hours and examined after a post-treatment interval of 5, 9 or 12 hours. Positive results were obtained 12 hours after exposure (Shimada et al. 1983). However, cell transformations were observed only at cytotoxic concentrations. Carcinogenic effects cannot be evaluated on the basis of this study.

A rat liver foci bioassay was carried out to investigate the tumour-promoting potential of 1,2,3-trichlorobenzene, 1,2,4-trichlorobenzene and 1,3,5-trichlorobenzene. In this assay, rat livers were examined for an increased incidence of gamma-glutamyltranspeptidase-positive foci (GGT foci) after initiation with diethylnitrosamine. A single intraperito­neal injection of 1,2,3-trichlorobenzene, 1,2,4-trichlorobenzene or 1,3,5-trichlorobenzene at a dose of 1.0 mmol/kg body weight (181 mg/kg body weight) was given to 7, 9 and 10 male and 2, 7 and 10 female Sprague Dawley rats, respectively, 1 and 5 weeks after initiation with diethylnitrosamine in a concentration of 0.5 mmol/kg body weight. The animals were sacrificed and examined 2 weeks after injection of the last dose. The incidence of GGT foci in the livers of the animals was not increased by exposure to any of the isomers (EC 2003; Henschler 1992; Herren-Freund and Pereira 1986). The design of the study makes it unsuitable for evaluating or disproving a tumour-promoting potential of trichlorobenzenes. Only 2 doses were given by intraperitoneal injection, a number too small to lead to promotion considering the half-lives of the trichlorobenzenes in the body.

#### 5.7.2 Long-term studies

##### 5.7.2.1 Oral administration

The data for the carcinogenic effects induced after oral administration are shown in Table 5 and 6.

In a 104-week feeding study, 1,2,4-trichlorobenzene (98.9% purity) was given to groups of 50 F344 rats per sex and dose. The study was carried out in compliance with OECD Test Guideline 451. The concentrations were 0, 150, 350 and 1200 mg/kg feed. These are equivalent to daily 1,2,4-trichlorobenzene doses of 0, 5.6, 19.4 or 66.5 mg/kg body weight for the male animals and 0, 6.9, 23.5 or 81.4 mg/kg body weight for the female animals. The highest dose was referred to as the maximum tolerable dose (MTD). In addition to the carcinogenic effects, mortality, body weights, feed consumption, haematological parameters and organ weights were examined. A full pathological and histopathological examination was carried out. Adverse, non-neoplastic effects were determined only in the animals of the high dose group (Section 5.5.2). In the high dose group, 60% of the male and 72% of the female animals survived. Mononuclear cell leukaemia and carcinomas in the pituitary gland and the Zymbal gland (histological examination carried out only if there were macroscopic findings) were observed in all groups and accounted for the mortality in all dose groups. The neoplastic findings were not regarded to be induced by the substance and their incidence was not increased with statistical significance in comparison with the incidences found in the control animals (ATSDR 2014; CMA 1994 a; EC 2003; Greim 2000; US EPA 2009 b). Mononuclear cell leukaemia and pituitary gland tumours also develop spontaneously in F344 rats, a strain that is susceptible for these effects (Maronpot et al. 2016). This explains the incidence of these tumours in all examined animals, including the control group. F344 rats have a low spontaneous incidence of tumours of the Zymbal gland. For this reason, a follow-up study was carried out to re-examine the development of this tumour form histopathologically. There was no evidence that the incidence of these tumours in the treated groups was increased with statistical significance in comparison with the incidence in the control group. A trend test was negative and Fisher’s test for significance yielded a value of p = 0.056 (Moore 2000 in EC (2003)). It is important to consider that humans do not have a Zymbal gland and that tumours that develop in this organ in rodent models after exposure to a non-genotoxic substance are not relevant for humans (Cohen 2004; Laube et al. 2019).

**Tab.5:** Carcinogenicity study in rats with 1,2,4-trichlorobenzene after oral administration

Author:	CMA 1994 b				
Substance:	1,2,4-trichlorobenzene (98.9% purity)				
Species:	**rat**, F344, 50 ♂, 50 ♀				
Administration route:	with the feed				
Concentration:	0, 100, 350 or 1200 mg/kg feed				
	♂: 0, 5.6, 19.4, 66.5 mg/kg body weight and day				
	♀: 0, 6.9, 23.5, 81.4 mg/kg body weight and day				
Duration:	104 weeks				
Toxicity:	mortality ↑, other adverse effects see Section 5.2.2				
		**Dose ♂/♀ [mg/kg body weight and day]**			
		**0**	**5.6/6.9^a)^**	**19.4/23.5^a)^**	**66.5/81.4**
surviving animals	♂	41/50 (82%)	39/49 (80%)	42/50 (84%)	30/50 (60%)
	♀	38/50 (76%)	37/49 (76%)	36/50 (72%)	36/50 (72%)
**neoplasms**					
**blood:**					
mononuclear cell leukaemia	♂	15/50 (30%)	13/49 (27%)	18/50 (36%)	21/50 (42%)
	♀	10/50 (20%)	10/49 (20%)	13/50 (26%)	10/50 (20%)
**Zymbal gland:**					
adenomas and carcinomas	♂	1/50 (2%)	0/49	1/50 (2%)	4/50 (8%)
	♀	0/50	2/49 (4%)^b)^	0/50	2/50 (4%)
both sexes combined	♂♀	1/100 (1%)	3/100 (3%)	1/100 (1%)	6/100 (6%)
**pituitary gland:**					
adenomas and carcinomas	♂	19/50 (38%)	13/49 (27%)	8/50 (16%)	12/50 (24%)
	♀	19/50 (38%)	22/49 (45%)	19/50 (38%)	21/50 (42%)

a) histological examination only if there were findings, all animals of the high dose group underwent histological examination

b) one of the tumours was characterized as an adenoma, all other tumours of the Zymbal gland were carcinomas; during the histopathological follow-up examination, all were evaluated as carcinomas (EC 2003) and another carcinoma was found in a female (total incidence 3/100)

In another 104-week feeding study, 1,2,4-trichlorobenzene (98.9% purity) was administered to 50 B6C3F1 mice per sex and dose. The study was carried out in accordance with OECD Test Guideline 451. The concentrations were 0, 150, 700 and 3200 mg/kg feed. These are equivalent to daily 1,2,4-trichlorobenzene doses of 0, 21, 100 or 520 mg/kg body weight for the male animals and 0, 26, 127 or 573 mg/kg body weight for the female animals. The highest dose was referred to as the MTD. In addition to the carcinogenic effects, mortality, body weights, feed consumption, haematological parameters and organ weights were examined. A full pathological and histopathological examination was performed. Adverse, non-neoplastic findings were determined in the dose groups (Section 5.5.2). At 520 and 573 mg/kg body weight fewer animals survived in comparison with the other groups; the difference was statistically significant. Likewise, the body weights and body weight gains were reduced with statistical significance in the animals of both sexes. Liver carcinomas were found in 100% of the males and 92% of the females, liver adenomas in 4% of the males and 15% of the females and centrilobular hepatocytomegaly in 40% of the males and 16% of the females. After exposure to 100 and 127 mg/kg body weight and day, liver carcinomas developed in 54% of the male animals and 56% of the female animals, liver adenomas in 32% of male mice and 32% of female mice and centrilobular hepatocytomegaly in 54% of the male animals and 2% of the female animals. In the groups treated with 21 and 26 mg/kg body weight and day, the incidences of adenomas or carcinomas were not increased with statistical significance compared with the control values. In the low dose group, liver adenomas formed in 14% of the males and 8% of the females and liver carcinomas in 10% and 2% of the animals, respectively. There was no evidence of an increased incidence of centrilobular hepatocytomegaly. However, these tumours occurred also in the control group: liver adenomas were found in 8% of the males and 6% of the females and liver carcinomas in 16% of the males and 2% of the females. None of the animals had tumours in organs or tissues other than the liver. The incidence of liver carcinomas increased with the dose and with the severity of the adverse effects observed in the liver; in the high dose group the incidence was very high. This effect was observed in 100% of the males and in 92% of the females. In the low dose group, the liver weights were increased, but not the tumour incidence (CMA 1994 a; Greim 2000). Limiting factors for the study were the high mortality in the high dose groups with survival of only 10% of the males and no surviving females. According to the authors, the early deaths of the animals were caused by the tumours in the liver. However, these findings must be evaluated with caution because of the use of the B6C3F1 mouse strain; this strain frequently develops liver tumours after exposure to hepatotoxic substances. In general, these neoplasms occur with a higher incidence in this strain (Laube et al. 2019; Maronpot 2009). The toxic effects on the liver caused by the trichlorobenzenes are described in Section 2 and Section 5.2 and may be closely associated with the formation of tumours.

**Tab.6:** Carcinogenicity study in mice with 1,2,4-trichlorobenzene after oral administration

Author:	CMA 1994 a				
Substance:	1,2,4-trichlorobenzene (98.9% purity)				
Species:	**mouse**, B6C3F1, 50 ♂, 50 ♀				
Administration route:	with the feed				
Concentration:	0, 150, 700 or 3200 mg/kg feed				
	♂: 0, 21, 100, 520 mg/kg body weight and day				
	♀: 0, 26, 127, 573 mg/kg body weight and day				
Duration:	104 weeks				
Toxicity:	mortality ↑, other adverse effects see Section 5.2.2				
		**Dose ♂/♀ [mg/kg body weight and day]**			
		**0**	**21/26**	**100/127**	**520/573**
surviving animals	♂	45/50 (90%)	44/49 (90%)	41/50 (82%)	5/50 (10%)
	♀	39/50 (78%)	37/49 (76%)	42/50 (84%)	0/50 (0%)
**tumours and preneoplasms**					
**liver:**					
liver adenomas	♂	4/50 (8%)	7/50 (14%)*	16/50 (32%)*	2/50 (4%)
	♀	3/50 (6%)	4/50 (8%)	16/50 (32%)*	8/50 (16%)*
liver carcinomas	♂	8/50 (16%)	5/50 (10%)	27/50 (54%)*	50/50 (100%)*
	♀	1/50 (2%)	1/50 (2%)	28/50 (56%)*	46/50 (92%)*
centrilobular hepatocytomegaly	♂	0/50	0/50	27/50 (54%)*	20/50 (40%)*
	♀	0/50	0/50	1/50 (2%)	8/50 (16%)*

*p < 0.05

##### 5.7.2.2 Dermal application

Over a period of 104 weeks, 0.03 ml of a 30% or 60% solution of 1,2,4-trichlorobenzene in acetone was applied twice a week to the dorsal skin of 75 Slc:ddY mice per sex and dose group. This is equivalent to doses of about 250 and 500 mg/kg body weight and day. The animals in the control groups were treated with acetone. The study did not include a group of animals that did not receive any form of treatment. The mortality in all groups of animals was very high, including the control groups. In week 83, survival among the treated animals was less than 10% in the males and less than 15% in the females. The causes of death were respiratory tract infections, amyloidosis of the liver, spleen and kidneys and tumour formation in the lungs, kidneys, stomach, bladder, the mamma and skin. The histopatho­logical examination revealed that more effects occurred in the animals of the high dose group than in the other groups; however, the number of animals examined was not specified. Likewise, the time points of tumour development were not reported. Tumours were found both in the treatment groups and in the control groups and were characterized as spontaneous and unrelated to the substance. Again, it was not specified whether the tumours were found in different animals or whether several tumours may have developed in individual animals. The skin tumours were characterized as squamous cell carcinomas, papillomas and fibromas (Henschler 1992; US EPA 2009 b; Yamamoto et al. 1982). This study has not been included in the evaluation because of methodological shortcomings, the high mortality and because the findings were reported in insufficient detail.

#### 5.7.3 Summary

Overall, the data available from long-term studies are not suitable for the evaluation of the carcinogenic effects of 1,2,3-trichlorobenzene and 1,3,5-trichlorobenzene. An in vitro cell transformation assay that investigated 1,2,4-trichlorobenzene in rat liver epithelial cells yielded positive findings only at cytotoxic concentrations and thus cannot be included in the evaluation. Rat liver foci studies with 1,2,4-trichlorobenzene cannot be used to rule out tumour-promoting effects because of the study design that was used. A study that investigated the carcinogenic effects of 1,2,4-trichlorobenzene after dermal application cannot be used to draw conclusions about these kinds of effects because its findings were not reported in sufficient detail and it had methodological shortcomings. In feeding studies with 1,2,4-trichlorobenzene in F344 rats and B6C3F1 mice, benign and malignant liver tumours developed in the mice at doses of 100 and 127 mg/kg body weight and day with an increased incidence that was statistically significant. In the feeding study with F344 rats, tumours of the Zymbal gland developed, a rare form of tumour for this rat strain, but the increased incidence was not statistically significant. Other neoplasms that occurred with an increased incidence were mononuclear cell leukaemia and tumours of the pituitary gland; however, the increase was not statistically significant. Overall, the evidence does not indicate that the tumours that formed in rats and mice are relevant for humans, as the incidence of liver adenomas and carcinomas was increased only in one species, a mouse strain that is known to be highly susceptible for this effect, and tumours did not form in other organs. Furthermore, the tumours of the Zymbal gland, a rare finding in rats, are likewise not considered relevant for humans in cases of exposure to non-genotoxic substances, such as the trichlorobenzenes. A lack of human relevance is supported by the fact that the observed mononuclear cell leukaemias in the F344 rats occur with a high spontaneous incidence in this strain and also the number of tumours of the pituitary gland in the exposed animals was not increased with statistical significance compared with that of the control group.

### 5.8 Other effects

Studies with V79 lung fibroblasts from the Chinese hamster found strong evidence that all 3 isomers induce marked cytotoxic effects in these cells. An NRU (neutral red uptake) inhibition assay revealed cytotoxicity at 1,2,3-trichlorobenzene, 1,2,4-trichlorobenzene and 1,3,5-trichlorobenzene concentrations of 0.1, 0.1 and 1 mg/ml, respectively. A test for colony forming ability (CFA test) detected cytotoxic effects at 1,2,3-trichlorobenzene, 1,2,4-trichlorobenzene and 1,3,5-trichlorobenzene concentrations of 0.24 (0.04 mg/ml), 0.23 (0.04 mg/ml) and > 4.1 mM (0.74 mg/ml), respectively. In a flow cytometry (FACScan) test, 1,2,3-trichlorobenzene, 1,2,4-trichlorobenzene and 1,3,5-trichlorobenzene were cytotoxic at 0.01, 0.01 and 0.1 mg/ml, respectively (Fratello et al. 1997). Another study confirmed the induction of cytotoxic effects in ovarian cells of the Chinese hamster after incubation with 1,2,4-trichlorobenzene in a concentration of 0.5 mg/ml (Garrett and Lewtas 1983). Evidence of cytotoxicity by 1,2,4-trichlorobenzene was likewise found in alveolar macrophages of rabbits, embryonal cells of Syrian hamsters and the fibroblasts of neonatal Balb-3T3 mice and humans (Garrett et al. 1983). Overall, these studies confirm that the isomers are highly cytotoxic in mammalian cells, as was observed earlier by studies using bacteria and yeast cells.

## 6 Manifesto (MAK value/classification)

The critical effects induced by the trichlorobenzenes are the adverse effects on the liver of mice and rats and on the kidneys of rats that were observed in animal studies. Rats are considered the most sensitive species.

**MAK value. **The isomers 1,2,3-trichlorobenzene, 1,2,4-trichlorobenzene and 1,3,5-trichlorobenzene have similar metabolic pathways. In the first step of metabolism, all 3 isomers form arene oxides which are then converted into polar phenolic intermediates. Various phase I enzymes of the liver are induced, some by all 3 isomers, but most strongly by 1,2,4-trichlorobenzene. The liver and the kidneys are the main target organs of all 3 isomers. According to the limited dataset available for acute toxicity and the findings of a developmental toxicity study (Black et al. 1988), 1,2,4-trichlorobenzene causes stronger toxic effects than the 2 other isomers. The 1,2,4-isomer induces the most marked porphyrinogenic effects, which is consistent with the finding that, of the 3 isomers, it is the strongest inducer of δ-aminolaevulinate synthetase. However, most of the studies available for evaluation studied only the 1,2,4-isomer. Due to the similarities in their metabolism and on the basis of the available data, the 3 isomers are evaluated together. This approach is based on a safety assessment which includes a MAK value for all 3 isomers based on the NOAEC of the most toxic isomer (1,2,4-trichlorobenzene). This procedure is used in particular because the data situation is not sufficient to derive independent limit values for 1,2,3- or 1,3,5-trichlorobenzene.

In a 90-day inhalation study with rats, the only substance-induced adverse effect of 1,2,4-trichlorobenzene was an increased excretion of uroporphyrin and coproporphyrin (disruption of porphyrin metabolism) at 10 ml/m^3^. The NOAEC was 3 ml/m^3^. According to the authors, this effect precedes the other adverse effects induced by the substance in studies with inhalation exposure or oral administration (Dow Chemical Company 1977). In another study in rats that used a similar study design, no further substance-related histopathological effects occurred in addition to the disruption of porphyrin metabolism upon exposure to 1,2,4-trichlorobenzene in a concentration of 30 ml/m^3^ (Kociba et al. 1981). Thus, it can be concluded that the disruption of porphyrin metabolism by 1,2,4-trichlorobenzene at 10 ml/m^3^ is not only an early-onset adverse effect, but also the most sensitive end point of the systemic toxicity induced by the substance in rats. Therefore, the MAK value is derived using the NOAEC of 3 ml/m^3^. This takes into account the increased respiratory volume at the workplace (1:2) and the extrapolation of the data from animal studies to humans (1:2). On the basis of the study of Kociba et al. (1981), an increase in severity after chronic exposure cannot be detected for this end point. Likewise, the inhalation study by Coate et al. (1977) did not find an intensification of the liver effects over time. On the basis of these findings, a value of 0.75 ml/m^3^ (5.6 mg/m^3^) was derived for 1,2,4-trichlorobenzene. After applying the preferred value approach, a MAK value of 0.5 ml/m^3^ (3.8 mg/m^3^) was derived for the trichlorobenzenes. The vapour pressures of 1,2,3-trichlorobenzene and 1,3,5-trichlorobenzene, which are solids, are so high that their MAK value can also be given in ml/m^3^. As the structurally related 1,4-dichlorobenzene has a MAK value of 2 ml/m^3^ but is a stronger irritant (Hartwig and MAK Commission 2019), the MAK value for the trichlorobenzenes based on systemic effects protects against irritation also at an excursion factor of 2. This assumption is supported also by inhalation studies with monkeys, rabbits and rats in which no effects on the respiratory tract occurred after exposure to 1,2,4-trichlorobenzene concentrations of 25 and 100 ml/m^3^ for 26 weeks (Coate et al. 1977). These effects were likewise not observed in rats exposed to 1,3,5-trichlorobenzene at 100 mg/m^3^ (about 13 ml/m^3^) for 13 weeks (Sasmore et al. 1983). Therefore, local effects on the respiratory tract are not expected to be induced by the trichlorobenzenes as long as the MAK value is not exceeded.

**Peak limitation. **As the MAK value was derived from systemic effects, the trichlorobenzenes have been assigned to Peak Limitation Category II. Available data on the half-lives of the trichlorobenzene isomers and their metabolites are considered insufficient. Therefore, the default excursion factor of 2 has been assigned. As stated above in the section on the MAK value, the trichlorobenzenes are not expected to induce irritation as long as this factor is not exceeded.

**Prenatal toxicity. **In a prenatal developmental toxicity study in Sprague Dawley rats with gavage administration, no toxic effects on development were observed up to the highest doses tested, which induced maternal toxicity. As a result, the NOAELs for developmental toxicity were 300 mg/kg body weight and day for 1,2,4-trichlorobenzene and 600 mg/kg body weight and day for 1,2,3-trichlorobenzene and 1,3,5-trichlorobenzene (Black et al. 1988; Greim 2007; Henschler 1992). A multi-generation study with exposure of Sprague Dawley rats via the drinking water likewise did not find evidence of perinatal toxicity up to the highest dose, which lead to parental toxicity. As a result, the NOAEL for perinatal toxicity induced by 1,2,4-trichlorobenzene was 54 mg/kg body weight and day. The following data are taken into consideration for the extrapolation of the NOAELs for developmental toxicity and perinatal toxicity to a concentration in workplace air: the daily exposure in the multi-generation study in comparison with the 5 days a week exposure at the workplace (7:5) and, with respect to both studies, the corresponding species-specific toxicokinetic correction value for the rat (1:4), the experimentally determined oral absorption (80%; ATSDR 2014), the body weight (70 kg) and respiratory volume (10 m^3^) of the person and the assumed 100% absorption by inhalation. The concentrations calculated from this are 840 mg/m^3^ (developmental toxicity of 1,2,3-trichlorobenzene and 1,3,5-trichlorobenzene), 420 mg/m^3^ (developmental toxicity of 1,2,4-trichlorobenzene) and 106 mg/m^3^ (perinatal toxicity of 1,2,4-trichlorobenzene). These result in 224-fold, 112-fold and 28-fold margins to the MAK value of 3.8 mg/m^3^. Due to the large margins between the extrapolated NOAELs and the MAK value as well as the lack of teratogenic effects, the 2 isomers 1,2,3-trichlorobenzene and 1,3,5-trichlorobenzene remain classified in Pregnancy Risk Group C. 1,2,4-Trichlorobenzene has likewise been assigned to this pregnancy risk group.

**Carcinogenicity. **After oral administration of 1,2,4-trichlorobenzene to B6C3F1 mice, benign and malignant tumours were found in the liver. These findings were observed only concurrently with marked liver toxicity and are not regarded as relevant for humans (see Section 2 and Section 5.7). The tumour incidence was not increased with statistical significance in F344 rats. The tumours of the Zymbal gland, which seldom occur spontaneously, are not regarded as relevant for humans in the case of non-genotoxic substances (Laube et al. 2019). The trichlorobenzenes were not found to be genotoxic in an overall review of the data. On the basis of all data available for carcinogenicity, the mechanism of action and the lack of genotoxic effects, 1,2,3-trichlorobenzene, 1,2,4-trichlorobenzene and 1,3,5-trichlorobenzene have not been assigned to a category for carcinogenic substances. The MAK value protects against the development of adverse effects in the liver and thus against subsequent tumour-promoting effects.

**Germ cell mutagenicity. **In valid studies, 1,2,3-trichlorobenzene, 1,2,4-trichlorobenzene and 1,3,5-trichloro­benzene were not mutagenic in bacteria and not clastogenic in mammalian cells in vitro as well as in vivo. Data for germ cells are not available. As a result, 1,2,3-trichlorobenzene, 1,2,4-trichlorobenzene and 1,3,5-trichlorobenzene have not been classified in a category for germ cell mutagens.

**Absorption through the skin. **There are no quantitative data available for the absorption of the 3 trichlorobenzene isomers through the skin. In animal studies, systemic toxic effects are induced after dermal application of 1,2,4-trichlorobenzene. Model calculations were carried out for 1,2,4-trichlorobenzene, the isomer that penetrates the skin most readily and has the highest toxicity, assuming standard conditions (1-hour exposure of 2000 cm^2^ of skin to a saturated aqueous solution). The models predicted absorption through the human skin in amounts of 12.7 mg and above. Assuming 100% absorption by inhalation and a respiratory volume of 10 m^3^, exposure to the systemically tolerable concentration of 5.6 mg/m^3^ would lead to the absorption of 56 mg of trichlorobenzene. Therefore, amounts far exceeding 25% of the systemically tolerable amount may be absorbed through the skin. As a result, 1,2,3-trichlorobenzene, 1,2,4-trichlorobenzene and 1,3,5-trichlorobenzene remain designated with an “H” (for substances which can be absorbed through the skin in toxicologically relevant amounts).

**Sensitization. **There are still no human data or positive findings from animal studies or from in vitro studies available for skin sensitization by 1,2,4-trichlorobenzene and 1,3,5-trichlorobenzene. Thus, they continue not to be designated with an “Sh” (for substances which cause sensitization of the skin). However, a valid LLNA found that 1,2,3-trichlorobenzene has skin sensitizing potential; for this reason, the substance has been designated with “Sh”. The indices for ear swelling and mass do not deviate significantly from the control values. Therefore, the positive results obtained in the LLNA for cell proliferation were apparently not adversely affected by the irritant effects of 1,2,3-trichlorobenzene. As there are no data for sensitizing effects on the respiratory tract, none of the 3 isomers have been given the “Sa” designation (for substances which cause sensitization of the airways).
